# Low Pathogenic Avian Influenza Exposure Risk Assessment in Australian Commercial Chicken Farms

**DOI:** 10.3389/fvets.2018.00068

**Published:** 2018-04-26

**Authors:** Angela Bullanday Scott, Jenny-Ann Toribio, Mini Singh, Peter Groves, Belinda Barnes, Kathryn Glass, Barbara Moloney, Amanda Black, Marta Hernandez-Jover

**Affiliations:** ^1^Sydney School of Veterinary Science, Faculty of Science, University of Sydney, Sydney, NSW, Australia; ^2^Quantitative Sciences, Department of Agriculture and Water Resources, Canberra, ACT, Australia; ^3^College of Medicine, Biology and Environment, Australian National University, Canberra, ACT, Australia; ^4^New South Wales Department of Primary Industries, Orange, NSW, Australia; ^5^Graham Centre for Agricultural Innovation, School of Animal and Veterinary Sciences, Charles Sturt University and New South Wales Department of Primary Industries, Wagga Wagga, NSW, Australia; ^6^School of Animal and Veterinary Sciences, Charles Sturt University, Wagga Wagga, NSW, Australia

**Keywords:** avian influenza, Australia, commercial chickens, scenario trees, exposure assessment, H5, H7

## Abstract

This study investigated the pathways of exposure to low pathogenic avian influenza (LPAI) virus among Australian commercial chicken farms and estimated the likelihood of this exposure occurring using scenario trees and a stochastic modeling approach following the World Organization for Animal Health methodology for risk assessment. Input values for the models were sourced from scientific literature and an on-farm survey conducted during 2015 and 2016 among Australian commercial chicken farms located in New South Wales and Queensland. Outputs from the models revealed that the probability of a first LPAI virus exposure to a chicken in an Australian commercial chicken farms from one wild bird at any point in time is extremely low. A comparative assessment revealed that across the five farm types (non-free-range meat chicken, free-range meat chicken, cage layer, barn layer, and free range layer farms), free-range layer farms had the highest probability of exposure (7.5 × 10^−4^; 5% and 95%, 5.7 × 10^−4^—0.001). The results indicate that the presence of a large number of wild birds on farm is required for exposure to occur across all farm types. The median probability of direct exposure was highest in free-range farm types (5.6 × 10^−4^ and 1.6 × 10^−4^ for free-range layer and free-range meat chicken farms, respectively) and indirect exposure was highest in non-free-range farm types (2.7 × 10^−4^, 2.0 × 10^−4^, and 1.9 × 10^−4^ for non-free-range meat chicken, cage layer, and barn layer farms, respectively). The probability of exposure was found to be lowest in summer for all farm types. Sensitivity analysis revealed that the proportion of waterfowl among wild birds on the farm, the presence of waterfowl in the range and feed storage areas, and the prevalence of LPAI in wild birds are the most influential parameters for the probability of Australian commercial chicken farms being exposed to avian influenza (AI) virus. These results highlight the importance of ensuring good biosecurity on farms to minimize the risk of exposure to AI virus and the importance of continuous surveillance of LPAI prevalence including subtypes in wild bird populations.

## Introduction

Low pathogenic avian influenza (LPAI) viruses are naturally circulating in wild birds globally. Birds in the taxonomic orders Anseriformes (waterfowl including ducks and geese) and Charadriiformes (shorebirds including gulls, waders, and auks) constitute the major natural reservoir of LPAI with an approximate prevalence of 2.5 and 0.6%, respectively, in Australia (1). Domestic gallinaceous (e.g., chickens and turkeys) poultry can become infected with LPAI *via* the fecal–oral route whereby poultry consume infectious fecal material from wild birds; either consuming feces directly or indirectly, such as through contaminated water, aerosol, or fomites. Once poultry are infected with LPAI, the virus may then mutate to highly pathogenic avian influenza (HPAI). During HPAI infection, morbidity and mortality rates in gallinaceous poultry are very high (50–89%) and can reach 100% in some flocks (2).

Highly pathogenic avian influenza (AI) is classed as a category 2 emergency animal disease in Australia under the Emergency Animal Disease Response Agreement as it has the potential to cause severe production losses and impact the national economy, and potentially impact human health and/or the environment (3). Australia has experienced seven HPAI outbreaks in poultry farms since 1976. All outbreaks were eradicated using a “stamping out” strategy which involved quarantine and culling of all poultry on infected premises, tracing and surveillance of farms at risk and restriction of movement to reduce spread of the virus (4–6). To date HPAI virus has not been detected in wild birds in Australia (1).

There is concern from experts within the Australian poultry industry about the change in probability of AI outbreak occurrence with the recent consumer driven expansion of free-range poultry farms. There are approximately 800 commercial contract meat chicken grower farms and 300 commercial chicken egg farms currently in Australia (7, 8). Products from commercial chicken farms account for the large majority of the national market; where the top seven meat chicken companies and the top 50 chicken egg producers supply over 95 and 80% of the national chicken meat and eggs consumed in Australia (7–9). Non-commercial chicken farms are small-scale farms that are suspected or proven to have less adoption of biosecurity practices than commercial farms. However, there is limited contact between non-commercial and commercial chicken farms and so the risk of exposure to disease on non-commercial farms may be higher but they do not appear to be a threat to the Australian chicken industry. The cost of a disease outbreak in non-commercial farms would also be less than commercial farms due fewer birds to destroy and a small overall impact on the industry, market and consumers (10). New South Wales (NSW) is the leading state for both egg and chicken meat production; producing 32 and 34% of the national egg and chicken meat volumes, respectively. Queensland and Victoria follow, producing 28 and 27% of the national egg volume, respectively, and 19 and 24% of the national chicken meat volume, respectively (7–9). The highest concentrations of egg farms in the country are in the Greater Sydney (31%) and Hunter regions (20%) (9). Free-range chicken meat production in Australia was regarded as a “cottage industry” in 2006 and now accounts for at least 15% of the total market (7). Similarly, the retail market share of free-range eggs has increased from 10% in 2000 to 50% in 2017 (8). The concern is that the expansion of free-range poultry farms will increase the opportunities of contact between wild birds and domestic poultry in Australia, thus potentially increasing the probability of LPAI exposure in Australian commercial chicken farms.

There are substantial differences in farm design, management, and biosecurity practices among the Australian commercial chicken enterprises, i.e., cage, barn, and free-range systems of both layer and meat chicken farms which can influence wild bird presence on farm (11, 12). In addition, previous work has identified differences in the type of wild birds present on farms of different production types and biosecurity implementation (13). There is a need to quantify and compare the probability of LPAI exposure for all types of Australian commercial chicken enterprises considering these differences. In addition, there is a need to investigate the effect of on-farm preventive actions that can mitigate the risk and impact of future AI outbreak occurrences in Australia. The aim of this study was to consider the potential pathways for LPAI exposure from wild birds to chickens present on all types of commercial chicken enterprises in Australia (non-free-range meat, free-range meat, cage layer, barn layer, free-range layer), and then to conduct a comparative assessment of how likely LPAI exposure from wild birds to chickens would occur via each of the considered pathways and overall for each farm type. The most influential pathways of exposure are also identified, thereby leading to recommendations about on-farm biosecurity practices that could be implemented to minimize these risks.

## Materials and Methods

### Risk Assessment Model

The World Organisation of Animal Health provides a methodological framework for conducting animal health risk analysis (14). Risk assessment is a component of the overall risk analysis methods, which involves an entry, exposure and consequence assessment, and an estimation of the risk. The current study uses an exposure assessment to investigate the potential exposure to AI viruses of domestic chickens raised in commercial chicken properties in Australia. A partial consequence assessment was also conducted and is presented in a subsequent paper (15). The exposure assessment considers all the potential pathways by which chickens located in a commercial layer or meat chicken farm can be exposed to AI virus from wild birds and the probability of these pathways occurring is calculated. Such pathways were portrayed using scenario trees (16) and developed using Microsoft Excel (PC/Windows 7, 2010). The probabilities were estimated using Monte Carlo stochastic simulation modeling using the program @RISK 7.0 (Palisade Corporation, USA) implemented in Microsoft Excel. Each simulation consisted of 50,000 iterations sampled using the Latin hypercube method with a fixed random seed of one.

### Data Sources

Most of the input values used in this model were parameterized using data collected from a survey on commercial chicken farms in Australia (11, 12). The survey defined commercial layer farms as those with more than 1,000 birds and commercial meat chicken farms as those with more than 25,000 birds. It involved a comprehensive on-farm interview with farmers, including questions related to farm management, biosecurity practices, and wild bird presence. Scientific literature was used to estimate input parameters when required.

### Survey on Commercial Layer and Meat Chicken Farms in the Sydney Basin Region and South East Queensland

A survey commenced in mid-2015 involving on-farm interviews with the farm manager or farm owner on 73 commercial chicken farms; 15 non-free-range meat, 15 free-range meat, nine cage layer, 9 barn layer, and 25 free-range layer farms. The farms were located in the Sydney basin region in NSW and in South East Queensland. The Sydney basin region was selected due to the high concentration of both layer and meat chicken farms in this area. However in this region, free-range meat chicken farms are all owned by one of two large privately owned meat chicken companies in Australia. Therefore, additional farm visits to South East Queensland were conducted to gain more representative data of privately owned meat chicken companies in Australia. The interviews involved a comprehensive questionnaire which asked questions to the farmers relating to biosecurity practices performed on farm, wild bird and animal presence, general farm information, and farm management. A greater proportion of layer farms and of free-range farms were surveyed due to the greater perceived risk of AI occurrence on these farm types. More details on the methodology of the survey, including the region and farm selection, questionnaire development, and conduct of the on-farm interviews, can be found in Scott et al. (11).

### Statistical Analysis

The statistical program JMP^®^ was used (© 2012 SAS Institute Inc., Cary, NC, USA) to conduct one-way analysis of variance (ANOVA) to analyze differences between the outcome probabilities for each of the different farm types. The outcome probabilities compared using ANOVA were the outcome probability from 1,000 iterations of the exposure scenario tree model simulation for each farm type, with each iteration reflecting the situation for one farm at a point in time. A *p*-value of <0.05 was used to determine statistical significance in these analyses.

### Probability of Exposure

The exposure assessment examines all potential pathways by which AI virus can be introduced from wild birds into a commercial layer or meat chicken farm and estimates the probability that a first exposure to a chicken occurs through each of these pathways. Five exposure assessments were performed, one for each farm type: non-free-range meat chicken farms, free-range meat chicken farms, cage layer farms, barn layer farms, and free-range layer farms. Only LPAI viruses were considered due to the fact that HPAI viruses have never been detected from Australian wild birds during surveillance activities (1).

In addition, the models considered differences depending on the season or time of the year, given virus prevalence in wild birds changes during different times of the year. The probability of chickens accessing outdoors in free-range farms also changes during different times of the year due to weather conditions. Therefore, three “seasons” were considered in the exposure assessments; winter (June–August), summer (December–February), and then autumn and spring (March–May and September–November) were combined as one season.

Parameters required in these exposure assessments included (1) the probability of wild bird presence in different areas of the commercial chicken farm; (2) the probability of wild birds being infected and excreting LPAI viruses; and (3) parameters in relation to the management and biosecurity practices performed on the farm that would increase or reduce the probability of exposure. The main pathways of exposure considered in these assessments were the following six pathways: (1) direct exposure in a shed; (2) direct exposure around feed storage areas; (3) indirect exposure through fomites or vectors; (4) indirect exposure through aerosol; (5) indirect exposure through contaminated water inside; (6) indirect exposure through contaminated water outside sheds; and (7) direct exposure on the range.

For the purpose of this model, direct exposure is defined as physical contact between a wild bird and a commercial chicken or direct contact between a commercial chicken and wild bird feces. By contrast, indirect exposure is defined as a commercial chicken coming into contact with the virus through a medium, i.e., through water, fomites, or vectors. Fomites include boots and equipment contaminated with wild bird feces and are subsequently in contact with chickens through movement. Biological vectors may become infected with the virus, most notably insects, mice, and rats, and may shed the virus in the presence of chickens or be consumed by chickens. Mechanical vectors, such as dogs and cats, can also present the virus to chickens through movement only.

The overall probability of exposure represents the probability of a first exposure to a domestic chicken by one wild bird in each farm type, irrespective of the pathway of exposure. This probability was calculated by summing the outcome probability of all the pathways that lead to exposure for each farm type. In addition, the overall probability of direct and indirect exposure was calculated by summing the outcome probabilities of the direct (pathways 1, 2, and 7) and indirect (pathways 3, 4, 5, and 6) pathways, respectively, which lead to exposure for each farm type.

The models estimate the probability of exposure posed by a single wild bird at any point in time. This outcome probability of exposure is then used to estimate the expected number of exposures considering a range of the number of wild birds that could visit a property at any point in time and using binomial distributions. The standard prevalence of LPAI, at approximately 2.5, 0.6, and 0.5% for waterfowl, shorebirds, and other bird types, respectively, of which a subset are H5 and H7 subtypes, was used for these binomial distributions (1). The prevalence of LPAI in waterfowl and the proportion of waterfowl on the farm was then also changed in the model to assess the expected number of exposures in potential worst-case scenarios: (1) 100% waterfowl proportion and no change in waterfowl LPAI prevalence; (2) 80% waterfowl proportion and 20% waterfowl LPAI prevalence; (3) 100% waterfowl proportion and 10% waterfowl LPAI prevalence; (4) 50% waterfowl proportion and 20% waterfowl LPAI prevalence; and (5) 50% waterfowl proportion and 10% waterfowl LPAI prevalence. The distributions assume that all wild birds are independent, have the same probability of being infected, and those infected are always infective.

**Figure 1:**
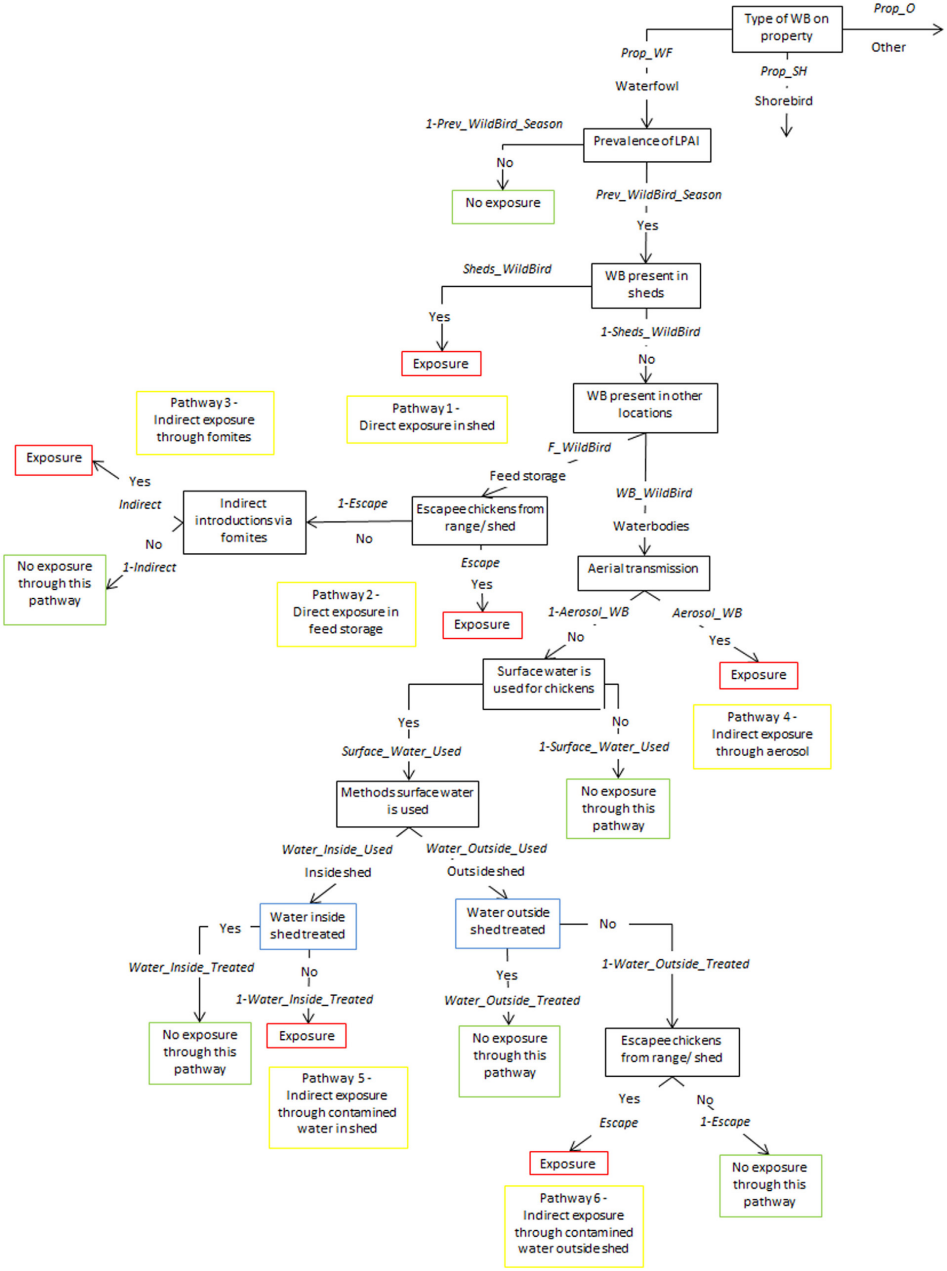
Scenario tree representing the exposure of chickens on non-free-range layer farms to low pathogenic avian influenza (LPAI) viruses from wild birds in Australia (*Prop_WF*, proportion of waterfowl answers reported on property, *Prop_SH*, proportion of shorebird answers reported on property, *Prop_O*, proportion of other bird types reported on property, *Prev_WildBird_Season*, prevalence of LPAI of the respective wild bird type (waterfowl, shorebird, or other) in the respective season (winter, summer, or autumn/spring), *Sheds_WildBird*, proportion of farms that reported the respective wild bird type (waterfowl, shorebird, or other) inside chicken sheds on the farm, *F_WildBird*, proportion of answers that witnessed the respective wild bird type (waterfowl, shorebird, or other) in feed storage areas, *WB_WildBird*, proportion of answers that witnessed the respective wild bird type (waterfowl, shorebird, or other) in waterbodies on/near the farm, *Escape*, proportion of farms that reported chickens escaping from shed, *Indirect*, probability of the occurrence of other indirect methods that can introduce LPAI (boots, mice/rats, insects, farm cats, or dogs), *Aerosol_WB*, probability of LPAI exposure from aerial dispersion of virus from wild birds on waterbodies, *Surface_Water_Used*, proportion of answers that surface water is used for chicken farm, *Water_Inside_Used*, proportion of answers that surface water is used inside sheds, *Water_Outside_Used*, proportion of answers that surface water is used outside sheds, *Water_Inside_Treated*, proportion of answers that treat water used inside sheds, *Water_Outside_Treated*, proportion of answers that treat water used outside sheds).

**Figure 2:**
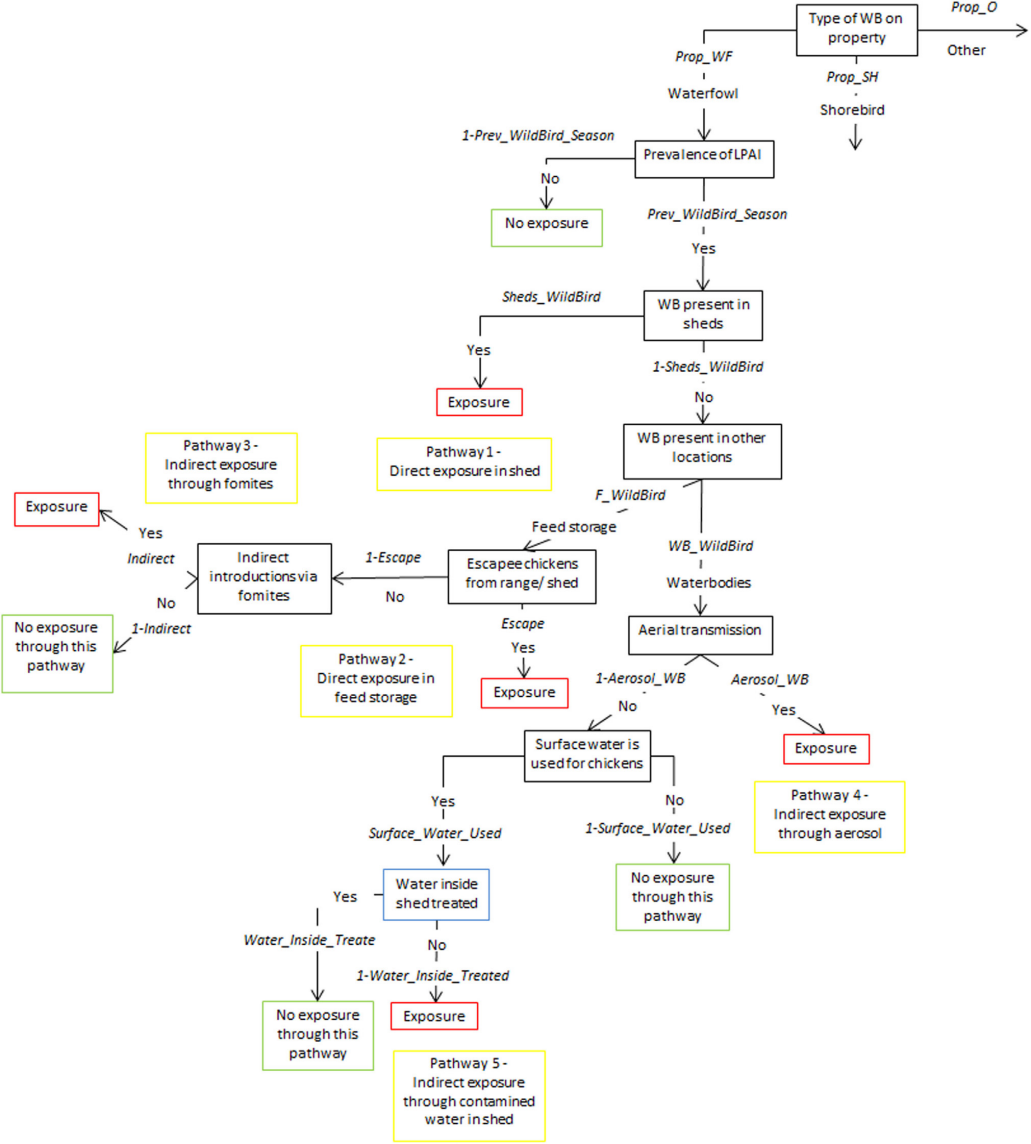
Scenario tree representing the exposure of chickens on non-free-range meat chicken farms to low pathogenic avian influenza (LPAI) viruses from wild birds in Australia (*Prop_WF*, proportion of waterfowl answers reported on property, *Prop_SH*, proportion of shorebird answers reported on property, *Prop_O*, proportion of other bird types reported on property, *Prev_WildBird_Season*, prevalence of LPAI of the respective wild bird type (waterfowl, shorebird, or other) in the respective season (winter, summer, or autumn/spring), *Sheds_WildBird*, proportion of farms that reported the respective wild bird type (waterfowl, shorebird, or other) inside chicken sheds on the farm, *F_WildBird*, proportion of answers that witnessed the respective wild bird type (waterfowl, shorebird, or other) in feed storage areas, *WB_WildBird*, proportion of answers that witnessed the respective wild bird type (waterfowl, shorebird, or other) in waterbodies on/near the farm, *Escape*, proportion of farms that reported chickens escaping from shed, *Indirect*, probability of the occurrence of other indirect methods that can introduce LPAI (boots, mice/rats, insects, farm cats, or dogs), *Aerosol_WB*, probability of LPAI exposure from aerial dispersion of virus from wild birds on waterbodies, Surface_Water_Used, proportion of answers that surface water is used for chicken farm, *Water_Inside_Treated*, proportion of answers that treat water used inside sheds).

**Figure 3:**
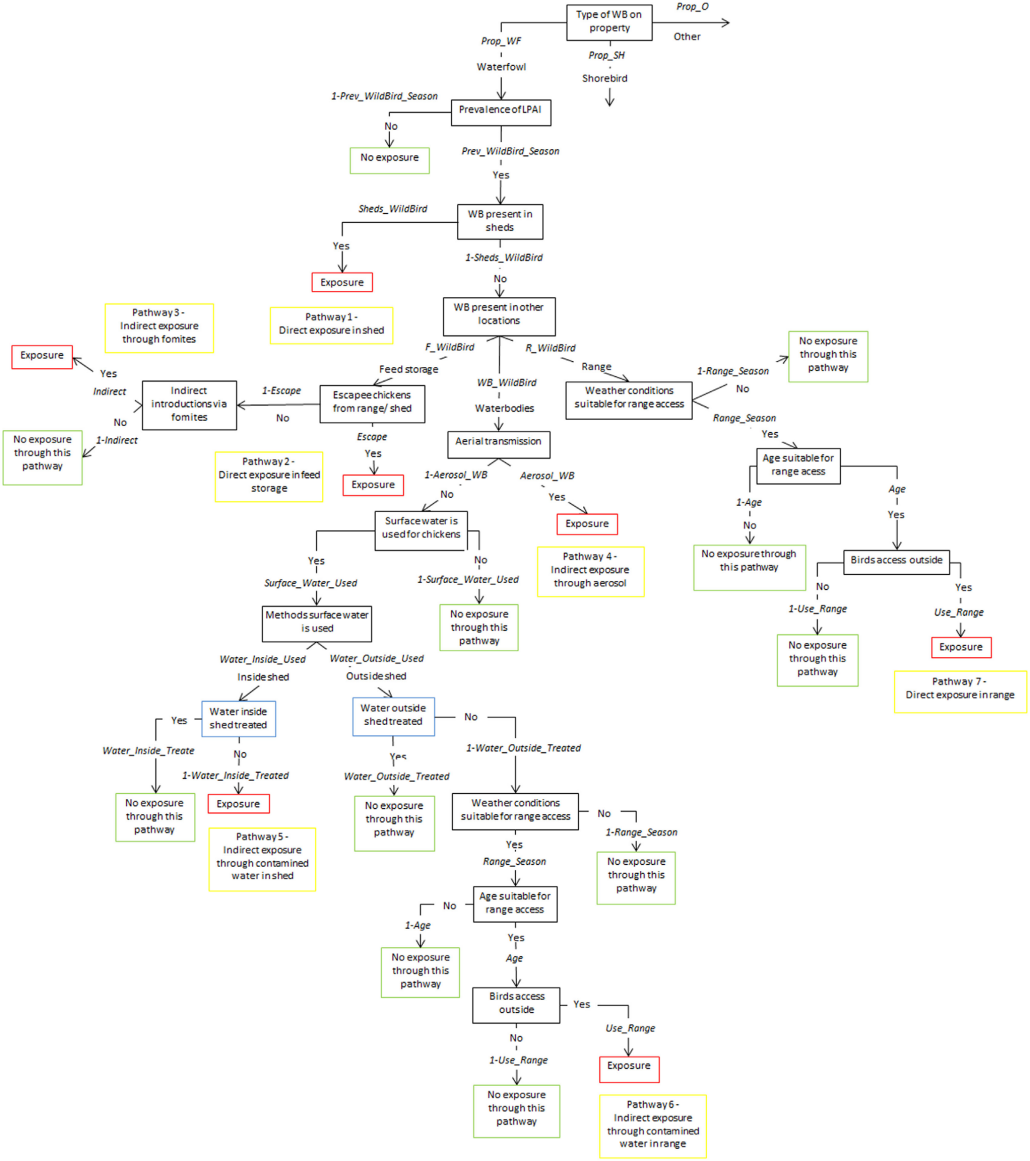
Scenario tree representing the exposure of chickens on free-range layer and meat chicken farms to low pathogenic avian influenza (LPAI) viruses from wild birds in Australia (*Prop_WF*, proportion of waterfowl answers reported on property, *Prop_SH*, proportion of shorebird answers reported on property, *Prop_O*, proportion of other bird types reported on property, *Prev_WildBird_Season*, prevalence of LPAI of the respective wild bird type (waterfowl, shorebird, or other) in the respective season (winter, summer, or autumn/spring), *Sheds_WildBird*, proportion of farms that reported the respective wild bird type (waterfowl, shorebird, or other) inside chicken sheds on the farm, *F_WildBird*, proportion of answers that witnessed the respective wild bird type (waterfowl, shorebird, or other) in feed storage areas, *WB_WildBird*, proportion of answers that witnessed the respective wild bird type (waterfowl, shorebird, or other) in waterbodies on/near the farm, *R_WildBird*, proportion of answers that witnessed the respective wild bird type (waterfowl, shorebird, or other) on the range, *Escape*, proportion of farms that reported chickens escaping from shed and from range, *Indirect*, probability of the occurrence of other indirect methods that can introduce LPAI (boots, mice/rats, insects, farm cats, or dogs), *Aerosol_WB*, probability of LPAI exposure from aerial dispersion of virus from wild birds on waterbodies, Surface_Water_Used, proportion of answers that surface water is used for chicken farm, *Water_Inside_Used*, proportion of answers that surface water is used inside sheds, *Water_Outside_Used*, proportion of answers that surface water is used outside sheds, *Water_Inside_Treated*, proportion of answers that treat water used inside sheds, *Water_Outside_Treated*, proportion of answers that treat water used outside sheds, *Range_Season*, probability that weather conditions are suitable for chickens to access range, *Age*, proportion of chicken’s lifetime in which they are allowed onto range, *Use_range*, proportion of flock that actually leave shed and use range).

Tables 1–5 provide a description of the nodes and input parameters of the scenario trees used for the exposure assessments for non-free-range meat chicken farms, free-range meat chicken farms, cage layer farms, barn layer farms, and free-range layer farms, respectively. Cage and barn layer farms are referred to as non-free-range layer farms and have the same scenario tree structure. Similarly, free-range layer and free-range meat chicken farms also have the same scenario tree structure. Therefore, the scenario trees used for non-free-range layer farms, non-free-range meat chicken farms, and free-range layer and meat chicken farms are depicted in Figures 1–3, respectively. Nodes and input parameters related to the range areas are specific for free-range farm types, and the nodes for pathways in which surface water is used are specific for layer farm types and free-range meat chicken farms. The pathway (6) indirect exposure through contaminated water outside sheds does not exist for non-free-range meat chicken farms, and pathway (7) direct exposure on the range does not exist for non-free-range meat chicken and layer farms. Detailed and further descriptions of the nodes are provided in the supplementary information.

**Table 1:** Nodes, parameter estimates, and input values used for the exposure assessment estimating the probability of exposure of commercial chickens on non-free-range meat chicken farms in Australia (specifically in the Sydney basin region and South East Queensland) to low pathogenic avian influenza (LPAI) from wild birds.

Node	Branch of node	Parameter estimates	Input values	Data sources
1. Type of wild bird on farm property	Waterfowl Shorebirds Other	Proportion of answers from farmers that reported the respective wild bird type on their property (*Prop_WF; Prop_SH; Prop_O*)	Beta (*s* + 1, *n* − *s* + 1) 48 answers of wild birds on the property (*n*); 16 answers of waterfowl; 7 answers of shorebirds; and 25 answers of other wild birds	Scott et al. (11, 13)

2. Prevalence of LPAI in wild birds	Yes No	Probability of the different wild bird types; waterfowl, shorebirds, or other, being infected with LPAI of H5 or H7 subtype in winter, summer, and autumn/spring (*Prev_WF_Winter; Prev_WF_Summer; Prev_WF_AuSp; Prev_SH_Winter; Prev_SH_Summer; Prev_SH_AuSp; Prev_O_Winter; Prev_O_Summer; Prev_O_AuSp*)	Beta (*s* + 1, *n* − *s* + 1) multiplied by the proportion of H5 and H7 of total positive influenza A samples in New South Wales (NSW) for the seasons winter, summer, and autumn/spring Information on the values for waterfowl and shorebirds that informed the Beta distributions for the three seasons and the proportion of influenza A samples that are H5 and H7 subtypes can be obtained by contacting the corresponding author 1,552 other bird types samples (*n*), 1 positive other bird type sample (*s*); this Beta distribution used for all three seasons	Grillo et al. (1) Hansbro et al. (17) NSW NAIWB Surveillance unpublished data (2016)

3. Respective wild bird type has been reported inside chicken sheds	Yes No	Proportion of farms that witnessed the respective wild bird type inside chicken sheds on the farm. The data suggest the probability for waterfowl and shorebirds inside sheds is close to 0 and, therefore, a Pert distribution is used for these wild bird types (*Sheds_WF; Sheds_SH; Sheds_O*)	*Sheds_WF* = Pert (0, 0, 0.05) *Sheds_SH* = Pert (0, 0, 0.05) *Sheds_O* = Beta (*s* + 1, *n* − *s* + 1) 15 non-free-range meat chicken farms surveyed; 7 reported seeing other wild bird types in sheds	Scott et al. (11, 13)

4. Respective wild bird type has been reported in other locations on the farm	Waterbodies Feed storage	Proportion of answers from farmers that witnessed the respective wild bird type in the respective areas (*WB_WF, F_WF; WB_SH, F_SH; WB_O, F_O*)	Beta (*s* + 1, *n* − *s* + 1) 16 answers of waterfowl in other locations (*n*); 13 answers of waterfowl in waterbodies; and 3 answers of waterfowl in feed storage areas 7 answers of shorebirds in other locations (*n*); 6 answers of shorebirds in waterbodies; and 1 answer of shorebirds in feed storage areas 18 answers of other wild bird types in other locations (*n*); 9 answers of other bird types in waterbodies; and 9 answers of other bird types in feed storage areas	Scott et al. (11, 13)

5. Aerial transmission of LPAI from wild birds to domestic chickens	Yes No	Probability of LPAI introduction *via* aerial dispersion from wild birds on waterbodies to chickens on farm (*Aerosol_WB*)	Beta (*s* + 1, *n* − *s* + 1) 12 air samples tested at less than 100 m from 83 LPAI (H5N2)-infected swans (*n*), 0 positive air samples obtained	Jonges et al. (18)

6. Surface water is used for chickens	Yes No	Proportion of farms that use surface water for the chicken farm (*Surface_Water_Used*)	Beta (*s* + 1, *n* − *s* + 1) 15 farms surveyed (*n*), 1 farm used surface water for farm	Scott et al. (11, 13)

7. Water inside chicken sheds is treated	Yes No	Proportion of answers from farmers that treat water inside the chicken sheds (*Water_Inside_Treated*)	Beta (*s* + 1, *n* − *s* + 1) 32 answers of water use inside chicken sheds (*n*), 28 answers of water treated inside chicken sheds	Scott et al. (11, 13)

8. Chickens have escaped the shed	Yes No	Proportion of farms that have reported chickens unintentionally outside of the shed (*Escape*)	Beta (*s* + 1, *n* − *s* + 1) 15 farms surveyed (*n*), 0 farms reported chickens escaped shed	Scott et al. (11, 13)

9. Other indirect routes that can lead to LPAI introduction	Yes No	Probability that chickens will be exposed to LPAI virus *via* other indirect methods; boots, mice/rats, insects, and pets combined into one probability (*Indirect*) (Probability of exposure from boots + mice/rats + insects + pets)	*Probability of exposure from boots* (PrBoots) 1/25 answers did not use footbaths avian influenza (AI) virus survival on boots is 3/6 days, considered high probability of exposure PrBoots = (1/25) × [Uniform (0.7, 1)] *Probability of exposure from mice/rats* (PrMice) 10/25 answers had mice/rats in sheds 12 mice inoculated (*n*), 0 positive on virus isolation PrMice = (10/25) × [Beta (*s* + 1, *n* − *s* + 1)] *Probability of exposure from insects* (PrInsects) 14/25 answers had insects in sheds 171 insects tested (*n*), 73 positive on virus isolation PrInsects = (14/25) × [Beta (*s* + 1, *n* − *s* + 1)] *Probability of exposure from pets* (PrPets) 0/25 answers allowed pets in sheds AI virus survival on feces is 2/6 days, considered moderate probability of exposure PrBoots = (1/25) × [Uniform (0.3, 0.5)]	Scott et al. (11, 13) Achenbach and Bowen (19) Nielsen et al. (20) Tiwari et al. (21) Nazir et al. (22)

**Table 2:** Nodes, parameter estimates, and input values used for the exposure assessment estimating the probability of exposure of commercial chickens on free-range meat chicken farms in Australia (specifically in the Sydney basin region and South East Queensland) to low pathogenic avian influenza (LPAI) from wild birds.

Node	Branch of node	Parameter estimates	Input values	Data sources
1. Type of wild bird on farm property	Waterfowl Shorebirds Other	Proportion of answers from farmers that reported the respective wild bird type on their property (*Prop_WF; Prop_SH; Prop_O*)	Beta (*s* + 1, *n* − *s* + 1) 36 answers of wild birds on the property (*n*); 12 answers of waterfowl; 2 answers of shorebirds; and 22 answers of other wild birds	Scott et al. (11, 13)

2. Prevalence of LPAI in wild birds	Yes No	Probability of the different wild bird types; waterfowl, shorebirds, or other, being infected with LPAI of H5 or H7 subtype in winter, summer, and autumn/spring (*Prev_WF_Winter; Prev_WF_Summer; Prev_WF_AuSp; Prev_SH_Winter; Prev_SH_Summer; Prev_SH_AuSp; Prev_O_Winter; Prev_O_Summer; Prev_O_AuSp*)	Beta (*s* + 1, *n* − *s* + 1) multiplied by the proportion of H5 and H7 of total positive influenza A samples in New South Wales (NSW) for the seasons winter, summer, and autumn/spring Information on the values for waterfowl and shorebirds that informed the Beta distributions for the 3 seasons and the proportion of influenza A samples that are H5 and H7 subtypes can be obtained by contacting the corresponding author 1,552 other bird types samples (*n*), 1 positive other bird type sample (*s*); this Beta distribution used for all three seasons	Grillo et al. (1) Hansbro et al. (17) NSW NAIWB Surveillance unpublished data (2016)

3. Respective wild bird type has been reported inside chicken sheds	Yes No	Proportion of farms that witnessed the respective wild bird type inside chicken sheds on the farm. The data suggest the probability for waterfowl and shorebirds inside sheds is close to 0 and, therefore, a Pert distribution is used for these wild bird types (*Sheds_WF; Sheds_SH; Sheds_O*)	*Sheds_WF* = Pert (0, 0, 0.05) *Sheds_SH* = Pert (0, 0, 0.05) *Sheds_O* = Beta (*s* + 1, *n* − *s* + 1) 15 farms surveyed; 11 reported seeing other wild bird types in sheds	Scott et al. (11, 13)

4. Respective wild bird type has been reported in other locations on the farm	Waterbodies Feed storage	Proportion of answers from farmers that witnessed the respective wild bird type in the respective areas (*WB_WF, F_WF; WB_SH, F_SH; WB_O, F_O*)	Beta (*s* + 1, *n* − *s* + 1) 20 answers of waterfowl in other locations (*n*); 14 answers of waterfowl in waterbodies; 2 answers of waterfowl in feed storage areas; and 4 answers of waterfowl on the range 4 answers of shorebirds in other locations (*n*); 2 answers of shorebirds in waterbodies; 0 answer of shorebirds in feed storage areas, and 2 answers of shorebirds on the range 37 answers of other wild bird types in other locations (*n*); 10 answers of other bird types in waterbodies; 12 answers of other bird types in feed storage areas; and 15 answers of other bird types on the range	Scott et al. (11, 13)

5. Suitable weather conditions for range access	Yes No	Probability that the weather conditions for seasons winter, summer, and autumn/spring are suitable for farmers to allow chickens on the range; when conditions are dry, between 17 and 28 C and there is no severe weather (*Range_Winter, Range_Summer, Range_AuSp*) (Probability of suitable temperature + dry conditions + no severe weather)	Beta (*s* + 1, *n* − *s* + 1) Winter: 13,248 winter hours recorded (*n*), 1,555 winter hours >17°C; 1,755 winter hours where precipitation >1 mm; 114 severe weather events in NSW, 1 severe weather events in Sydney basin in winter Summer: 13,248 summer hours recorded (*n*), 6,231.5 summer hours <28°C; 8,098.5 summer hours where precipitation >1 mm; 114 severe weather events in NSW, 64 severe weather events in Sydney basin in summer Autumn/Spring: 26,352 autumn/spring hours recorded (*n*), 9,338.5 autumn/spring hours >17 C and <28 C; 3,960.5 autumn/spring hours where precipitation >1 mm; 114 severe weather events in NSW, 49 severe weather events in Sydney basin in autumn/spring	Bureau of Meterology (23)

6. Birds are a suitable age for range access	Yes No	Proportion of the chicken’*s* lifetime in which they are allowed onto the range (*Age*)	Beta (*s* + 1, *n* − *s* + 1) Average age at flock depopulation 43.25 days (*n*), age allowed outside 21 days	Scott et al. (11, 13)

7. Birds actually go onto the range	Yes No	Proportion of flock that actually leave shed and use the range as reported by farmers (*Use_Range*)	Average of 15 Beta functions (*s* + 1, *n* − *s* + 1) Total flock proportion 100 (*n*); proportion of flock that use range (9 varying answers)	Scott et al. (11, 13)

8. Aerial transmission of LPAI from wild birds to domestic chickens	Yes No	Probability of LPAI introduction *via* aerial dispersion from wild birds on waterbodies to chickens on farm (*Aerosol_WB*)	Beta (*s* + 1, *n* − *s* + 1) 12 samples tested at less than 100 m from 83 LPAI (H5N2) infected swans (*n*), 0 positive air samples obtained	Jonges et al. (18)

9. Surface water is used for chickens	Yes No	Proportion of answers from farmers that use surface water for the chicken farm (*Surface_Water_Used*)	Beta (*s* + 1, *n* − *s* + 1) 15 farms surveyed (*n*), 2 answers used surface water for farm	Scott et al. (11, 13)

10. Locations surface water is used for	Inside shed Outside shed	Proportion of answers from farmers that use surface water for inside the shed versus outside the shed (*Water_Inside_Used, Water_Outside_Used*)	Beta (*s* + 1, *n* − *s* + 1) 6 answers of surface water use (*n*), 4 answers use surface water inside shed, and 2 answers use surface water outside shed	Scott et al. (11, 13)

11. Water inside chicken sheds is treated	Yes No	Proportion of answers from farmers that treat water inside the chicken sheds (*Water_Inside_Treated*)	Beta (*s* + 1, *n* − *s* + 1) 34 answers of water use inside chicken sheds (*n*), 34 answers of water treated inside chicken sheds	Scott et al. (11, 13)

12. Water outside chicken sheds is treated	Yes No	Proportion of answers from farmers that treat water used outside the shed (*Water_Outside_Treated*)	Beta (*s* + 1, *n* − *s* + 1) 9 answers of water use outside chicken sheds (*n*), 8 answers of water treated outside chicken sheds	Scott et al. (11, 13)

13. Chickens have escaped the shed or range area	Yes No	Proportion of farms that have reported chickens unintentionally outside of the shed or range area (*Escape*)	Beta (*s* + 1, *n* − *s* + 1) 15 farms surveyed (*n*), 0 farms reported chickens escaped shed or range area	Scott et al. (11, 13)

14. Other indirect routes that can lead to LPAI introduction	Yes No	Probability that chickens will be exposed to LPAI virus via other indirect methods; boots, mice/rats, insects and pets combined into one probability (*Indirect*) (Probability of exposure from boots + mice/rats + insects + pets)	*Probability of exposure from boots* (PrBoots) 1/28 answers did not use footbaths AI virus survival on boots is 3/6 days, considered high probability of exposure PrBoots = (1/28) × [Uniform (0.7, 1)] *Probability of exposure from mice/rats* (PrMice) 12/28 answers had mice/rats in sheds 12 mice inoculated (*n*), 0 positive on virus isolation PrMice = (10/25) × [Beta (*s* + 1, *n* − *s* + 1)] *Probability of exposure from insects* (PrInsects) 13/28 answers had insects in sheds 171 insects tested (*n*), 73 positive on virus isolation PrInsects = (13/28) × [Beta (*s* + 1, *n* − *s* + 1)] *Probability of exposure from pets* (PrPets) 2/28 answers allowed pets in sheds on range area AI virus survival on feces is 2/6 days, considered moderate probability of exposure PrBoots = (2/28) × [Uniform (0.3, 0.5)]	Scott et al. (11, 13) Henzler et al. (24) Achenbach and Bowen (19) Nielsen et al. (20) Tiwari et al. (21) Nazir et al. (22)

**Table 3:** Nodes, parameter estimates and input values used for the exposure assessment estimating the probability of exposure of commercial chickens on cage layer farms in Australia (specifically in the Sydney basin region and South East Queensland) to low pathogenic avian influenza (LPAI) from wild birds.

Node	Branch of node	Parameter estimates	Input values	Data sources
1. Type of wild bird on farm property	Waterfowl Shorebirds Other	Proportion of answers from farmers that reported the respective wild bird type on their property (*Prop_WF; Prop_SH; Prop_O*)	Beta (*s* + 1, *n* − *s* + 1) 30 answers of wild birds on the property (*n*); 9 answers of waterfowl; 2 answers of shorebirds; and 19 answers of other wild birds	Scott et al. (11, 13)

2. Prevalence of LPAI in wild birds	Yes No	Probability of the different wild bird types; waterfowl, shorebirds or other, being infected with LPAI of H5 or H7 subtype in winter, summer and autumn/spring (*Prev_WF_Winter; Prev_WF_Summer; Prev_WF_AuSp; Prev_SH_Winter; Prev_SH_Summer; Prev_SH_AuSp; Prev_O_Winter; Prev_O_Summer; Prev_O_AuSp*)	Beta (*s* + 1, *n* − *s* + 1) multiplied by the proportion of H5 and H7 of total positive influenza A samples in New South Wales (NSW) for the seasons winter, summer, and autumn/spring Information on the values for waterfowl and shorebirds that informed the Beta distributions for the 3 seasons and the proportion of influenza A samples that are H5 and H7 subtypes can be obtained by contacting the corresponding author 1,552 other bird types samples (*n*), 1 positive other bird type sample (*s*); this Beta distribution used for all three seasons	Grillo et al. (1) Hansbro et al. (17) NSW NAIWB Surveillance unpublished data (2016)

3. Respective wild bird type has been reported inside chicken sheds	Yes No	Proportion of farms that witnessed the respective wild bird type inside chicken sheds on the farm. The data suggests the probability for waterfowl and shorebirds inside sheds is close to 0 and, therefore, a Pert distribution is used for these wild bird types (*Sheds_WF; Sheds_SH; Sheds_O*)	*Sheds_WF* = Pert (0, 0, 0.05) *Sheds_SH* = Pert (0, 0, 0.05) *Sheds_O* = Beta (*s* + 1, *n* − *s* + 1) 9 farms surveyed; 5 reported seeing other wild bird types in sheds	Scott et al. (11, 13)

4. Respective wild bird type has been reported in other locations on the farm	Waterbodies Feed storage	Proportion of answers from farmers that witnessed the respective wild bird type in the respective areas (*WB_WF, F_WF; WB_SH, F_SH; WB_O, F_O*)	Beta (*s* + 1, *n* − *s* + 1) 9 answers of waterfowl in other locations (*n*); 9 answers of waterfowl in waterbodies; and 0 answers of waterfowl in feed storage areas 2 answers of shorebirds in other locations (*n*); 2 answers of shorebirds in waterbodies; and 0 answer of shorebirds in feed storage areas 14 answers of other wild bird types in other locations (*n*); 6 answers of other bird types in waterbodies; and 8 answers of other bird types in feed storage areas	Scott et al. (11, 13)

5. Aerial transmission of LPAI from wild birds to domestic chickens	Yes No	Probability of LPAI introduction via aerial dispersion from wild birds on waterbodies to chickens on farm (*Aerosol_WB*)	Beta (*s* + 1, *n* − *s* + 1) 12 samples tested at less than 100 m from 83 LPAI (H5N2) infected swans (*n*), 0 positive air samples obtained	Jonges et al. (18)

6. Surface water is used for chickens	Yes No	Proportion of farms that use surface water for the chicken farm (*Surface_Water_Used*)	Beta (*s* + 1, *n* − *s* + 1) 9 farms surveyed (*n*), 2 farm used surface water for farm	Scott et al. (11, 13)

7. Locations surface water is used for	Inside shed Outside shed	Proportion of answers from farmers that use surface water for inside the shed versus outside the shed (*Water_Inside_Used, Water_Outside_Used*)	Beta (*s* + 1, *n* − *s* + 1) 3 answers of surface water use (*n*), 1 answers use surface water inside shed, and 2 answers use surface water outside shed	Scott et al. (11, 13)

8. Water inside chicken sheds is treated	Yes No	Proportion of answers from farmers that treat water inside the chicken sheds (*Water_Inside_Treated*)	Beta (*s* + 1, *n* − *s* + 1) 18 answers of water use inside chicken sheds (*n*), 17 answers of water treated inside chicken sheds	Scott et al. (11, 13)

9. Water outside chicken sheds is treated	Yes No	Proportion of answers from farmers that treat water used outside the shed (*Water_Outside_Treated*)	Beta (*s* + 1, *n* − *s* + 1) 5 answers of water use outside chicken sheds (*n*), 2 answers of water treated outside chicken sheds	Scott et al. (11, 13)

10. Chickens have escaped the shed	Yes No	Proportion of farms that have reported chickens unintentionally outside of the shed (*Escape*)	Beta (*s* + 1, *n* − *s* + 1) 9 farms surveyed (*n*), 1 farms reported chickens escaped shed	Scott et al. (11, 13)

11. Other indirect routes that can lead to LPAI introduction	Yes No	Probability that chickens will be exposed to LPAI virus via other indirect methods; boots, mice/rats, insects and pets combined into one probability (*Indirect*) (Probability of exposure from boots + mice/rats + insects + pets)	*Probability of exposure from boots* (PrBoots) 7/30 answers did not use footbaths AI virus survival on boots is 3/6 days, considered high probability of exposure PrBoots = (7/30) × [Uniform (0.7, 1)] *Probability of exposure from mice/rats* (PrMice) 8/30 answers had mice/rats in sheds 12 mice inoculated (*n*), 0 positive on virus isolation PrMice = (10/25) × [Beta (*s* + 1, *n* − *s* + 1)] *Probability of exposure from insects* (PrInsects) 9/30 answers had insects in sheds 171 insects tested (*n*), 73 positive on virus isolation PrInsects = (9/30) × [Beta (*s* + 1, *n* − *s* + 1)] *Probability of exposure from pets* (PrPets) 6/30 answers allowed pets in sheds AI virus survival on feces is 2/6 days, considered moderate probability of exposure PrBoots = (6/30) × [Uniform (0.3, 0.5)]	Scott et al. (11, 13) Henzler et al. (24) Achenbach and Bowen (19) Nielsen et al. (20) Tiwari et al. (21) Nazir et al. (22)

**Table 4:** Nodes, parameter estimates, and input values used for the exposure assessment estimating the probability of exposure of commercial chickens on barn layer farms in Australia (specifically in the Sydney basin region and South East Queensland) to low pathogenic avian influenza (LPAI) from wild birds.

Node	Branch of node	Parameter estimates	Input values	Data sources
1. Type of wild bird on farm property	Waterfowl Shorebirds Other	Proportion of answers from farmers that reported the respective wild bird type on their property (*Prop_WF; Prop_SH; Prop_O*)	Beta (*s* + 1, *n* − *s* + 1) 26 answers of wild birds on the property (*n*); 7 answers of waterfowl; 2 answers of shorebirds; and 17 answers of other wild birds	Scott et al. (11, 13)

2. Prevalence of LPAI in wild birds	Yes No	Probability of the different wild bird types; waterfowl, shorebirds or other, being infected with LPAI of H5 or H7 subtype in winter, summer, and autumn/spring (*Prev_WF_Winter; Prev_WF_Summer; Prev_WF_AuSp; Prev_SH_Winter; Prev_SH_Summer; Prev_SH_AuSp; Prev_O_Winter; Prev_O_Summer; Prev_O_AuSp*)	Beta (*s* + 1, *n* − *s* + 1) multiplied by the proportion of H5 and H7 of total positive influenza A samples in New South Wales (NSW) for the seasons winter, summer, and autumn/spring Information on the values for waterfowl and shorebirds that informed the Beta distributions for the 3 seasons and the proportion of influenza A samples that are H5 and H7 subtypes can be obtained by contacting the corresponding author 1,552 other bird types samples (*n*), 1 positive other bird type sample (*s*); this Beta distribution used for all three seasons	Grillo et al. (1) Hansbro et al. (17) NSW NAIWB Surveillance unpublished data (2016)

3. Respective wild bird type has been reported inside chicken sheds	Yes No	Proportion of farms that witnessed the respective wild bird type inside chicken sheds on the farm. The data suggests the probability for waterfowl and shorebirds inside sheds is close to 0 and, therefore, a Pert distribution is used for these wild bird types (*Sheds_WF; Sheds_SH; Sheds_O*)	*Sheds_WF* = Pert (0, 0, 0.05) *Sheds_SH* = Pert (0, 0, 0.05) *Sheds_O* = Beta (*s* + 1, *n* − *s* + 1) 9 barn layer farms surveyed; 5 reported seeing other wild bird types in sheds	Scott et al. (11, 13)

4. Respective wild bird type has been reported in other locations on the farm	Waterbodies Feed storage	Proportion of answers from farmers that witnessed the respective wild bird type in the respective areas (*WB_WF, F_WF; WB_SH, F_SH; WB_O, F_O*)	Beta (*s* + 1, *n* − *s* + 1) 7 answers of waterfowl in other locations (*n*); 7 answers of waterfowl in waterbodies; 0 answers of waterfowl in feed storage areas 2 answers of shorebirds in other locations (*n*); 2 answers of shorebirds in waterbodies; 0 answer of shorebirds in feed storage areas 12 answers of other wild bird types in other locations (*n*); 4 answers of other bird types in waterbodies; 8 answers of other bird types in feed storage areas	Scott et al. (11, 13)

5. Aerial transmission of LPAI from wild birds to domestic chickens	Yes No	Probability of LPAI introduction via aerial dispersion from wild birds on waterbodies to chickens on farm (*Aerosol_WB*)	Beta (*s* + 1, *n* − *s* + 1) 12 samples tested at less than 100 m from 83 LPAI (H5N2) infected swans (*n*), 0 positive air samples obtained	Jonges et al. (18)

6. Surface water is used for chickens	Yes No	Proportion of farms that use surface water for the chicken farm (*Surface_Water_Used*)	Beta (*s* + 1, *n* − *s* + 1) 9 farms surveyed (*n*), 3 farms used surface water for farm	Scott et al. (11, 13)

7. Locations surface water is used for	Inside shed Outside shed	Proportion of answers from farmers that use surface water for inside the shed versus outside the shed (*Water_Inside_Used, Water_Outside_Used*)	Beta (*s* + 1, *n* − *s* + 1) 6 total answers of surface water use water is used for (*n*), 5 answers for inside the shed, 1 answer for outside the shed	Scott et al. (11, 13)

8. Water inside chicken sheds is treated	Yes No	Proportion of answers from farmers that treat water inside the chicken sheds (*Water_Inside_Treated*)	Beta (*s* + 1, *n* − *s* + 1) 19 answers of water use inside chicken sheds (*n*), 18 answers of water treated inside chicken sheds	Scott et al. (11, 13)

9. Water outside chicken sheds is treated	Yes No	Proportion of answers from farmers that treat water used outside the shed (*Water_Outside_Treated*)	Beta (*s* + 1, *n* − *s* + 1) 1 answers of water use outside chicken sheds (*n*), 1 answers of water treated outside chicken sheds	Scott et al. (11, 13)

10. Chickens have escaped the shed	Yes No	Proportion of farms that have reported chickens unintentionally outside of the shed (*Escape*)	Beta (*s* + 1, *n* − *s* + 1) 9 farms surveyed (*n*), 0 farms reported chickens escaped shed	Scott et al. (11, 13)

11. Other indirect routes that can lead to LPAI introduction	Yes No	Probability that chickens will be exposed to LPAI virus via other indirect methods; boots, mice/rats, insects and pets combined into one probability (*Indirect*) (Probability of exposure from boots + mice/rats + insects + pets)	*Probability of exposure from boots* (PrBoots) 3/21 answers did not use footbaths AI virus survival on boots is 3/6 days, considered high probability of exposure PrBoots = (3/21) × [Uniform (0.7, 1)] *Probability of exposure from mice/rats* (PrMice) 8/21 answers had mice/rats in sheds 12 mice inoculated (*n*), 0 positive on virus isolation PrMice = (8/21) × [Beta (*s* + 1, *n* − *s* + 1)] *Probability of exposure from insects* (PrInsects) 9/21 answers had insects in sheds 143 insects tested (*n*), 114 positive on virus isolation PrInsects = (9/21) × [Beta (*s* + 1, *n* − *s* + 1)] *Probability of exposure from pets* (PrPets) 1/21 answers allowed pets in sheds AI virus survival on feces is 2/6 days, considered moderate probability of exposure PrBoots = (1/21) × [Uniform (0.3, 0.5)]	Scott et al. (11, 13) Henzler et al. (24) Achenbach and Bowen (19) Nielsen et al. (20) Tiwari et al. (21) Nazir et al. (22)

**Table 5:** Nodes, parameter estimates and input values used for the exposure assessment estimating the probability of exposure of commercial chickens on free-range layer farms (specifically in the Sydney basin region and South East Queensland) in Australia to low pathogenic avian influenza (LPAI) from wild birds.

Node	Branch of node	Parameter estimates	Input values	Data sources
1. Type of wild bird on farm property	Waterfowl Shorebirds Other	Proportion of answers from farmers that reported the respective wild bird type on their property (*Prop_WF; Prop_SH; Prop_O*)	Beta (*s* + 1, *n* − *s* + 1) 140 answers of wild birds on the property (*n*); 44 answers of waterfowl; 33 answers of shorebirds; 63 answers of other wild birds	Scott et al. (11, 13)

2. Prevalence of LPAI in wild birds	Yes No	Probability of the different wild bird types; waterfowl, shorebirds or other, being infected with LPAI of H5 or H7 subtype in winter, summer, and autumn/spring (*Prev_WF_Winter; Prev_WF_Summer; Prev_WF_AuSp; Prev_SH_Winter; Prev_SH_Summer; Prev_SH_AuSp; Prev_O_Winter; Prev_O_Summer; Prev_O_AuSp*)	Beta (*s* + 1, *n* − *s* + 1) multiplied by the proportion of H5 and H7 of total positive influenza A samples in New South Wales (NSW) for the seasons winter, summer, and autumn/spring Information on the values for waterfowl and shorebirds that informed the Beta distributions for the 3 seasons and the proportion of influenza A samples that are H5 and H7 subtypes can be obtained by contacting the corresponding author 1,552 other bird types samples (*n*), 1 positive other bird type sample (*s*); this Beta distribution used for all three seasons	Grillo et al. (1) Hansbro et al. (17) NSW NAIWB Surveillance unpublished data (2016)

3. Respective wild bird type has been reported inside chicken sheds	Yes No	Proportion of farms that witnessed the respective wild bird type inside chicken sheds on the farm. The data suggests the probability for waterfowl and shorebirds inside sheds is close to 0 and, therefore, a Pert distribution is used for these wild bird types (*Sheds_WF; Sheds_SH; Sheds_O*)	*Sheds_WF* = Pert (0, 0, 0.05) *Sheds_SH* = Pert (0, 0, 0.05) *Sheds_O* = Beta (*s* + 1, *n* − *s* + 1) 25 farms surveyed; 13 reported seeing other wild bird types in sheds	Scott et al. (11, 13)

4. Respective wild bird type has been reported in other locations on the farm	Waterbodies Feed storage	Proportion of answers from farmers that witnessed the respective wild bird type in the respective areas (*WB_WF, F_WF; WB_SH, F_SH; WB_O, F_O*)	Beta (*s* + 1, *n* − *s* + 1) 44 answers of waterfowl in other locations (*n*); 23 answers of waterfowl in waterbodies; 9 answers of waterfowl in feed storage areas, 12 answers of waterfowl on the range 33 answers of shorebirds in other locations (*n*); 12 answers of shorebirds in waterbodies; 9 answer of shorebirds in feed storage areas, 12 answers of shorebirds on the range 50 answers of other wild bird types in other locations (*n*); 14 answers of other bird types in waterbodies; 16 answers of other bird types in feed storage areas, 20 answers of other bird types on the range	Scott et al. (11, 13)

5. Suitable weather conditions for range access	Yes No	Probability that the weather conditions for seasons winter, summer, and autumn/spring are suitable for farmers to allow chickens on the range; when conditions are dry, between 17 and 28 C and there is no severe weather (*Range_Winter, Range_Summer, Range_AuSp*) (Probability of suitable temperature + dry conditions + no severe weather)	Beta (*s* + 1, *n* − *s* + 1) Winter: 13,248 winter hours recorded (*n*), 1,555 winter hours > 17C; 1,755 winter hours where precipitation > 1mm; 114 severe weather events in NSW, 1 severe weather events in Sydney basin in winter Summer: 13,248 summer hours recorded (*n*), 6,231.5 summer hours <28°C; 8,098.5 summer hours where precipitation >1 mm; 114 severe weather events in NSW, 64 severe weather events in Sydney basin in summer Autumn/Spring: 26,352 autumn/spring hours recorded (*n*), 9,338.5 autumn/spring hours >17°C and <28°C; 3,960.5 autumn/spring hours where precipitation >1 mm; 114 severe weather events in NSW, 49 severe weather events in Sydney basin in autumn/spring	Bureau of Meterology (23)

6. Birds are a suitable age for range access	Yes No	Proportion of the chicken’*s* lifetime in which they are allowed onto the range (*Age*)	Beta (*s* + 1, *n* − *s* + 1) Average age at flock depopulation 87.32 weeks (*n*), average age allowed outside 22.94 weeks	Scott et al. (11, 13)

7. Birds actually go onto the range	Yes No	Proportion of flock that actually leave shed and use the range as reported by farmers (*Use_Range*)	Average of 25 Beta functions (*s* + 1, *n* − *s* + 1) Total flock proportion 100 (*n*); proportion of flock that use range (25 varying answers)	Scott et al. (11, 13)

8. Aerial transmission of LPAI from wild birds to domestic chickens	Yes No	Probability of LPAI introduction via aerial dispersion from wild birds on waterbodies to chickens on farm (*Aerosol_WB*)	Beta (*s* + 1, *n* − *s* + 1) 12 air samples tested at less than 100 m from 83 LPAI (H5N2) infected swans (*n*), 0 positive air samples obtained	Jonges et al. (18)

9. Surface water is used for chickens	Yes No	Proportion of answers from farmers that use surface water for the chicken farm (*Surface_Water_Used*)	Beta (*s* + 1, *n* − *s* + 1) 25 farms surveyed (*n*), 6 answers used surface water for farm	Scott et al. (11, 13)

10. Locations surface water is used for	Inside shed Outside shed	Proportion of answers from farmers that use surface water for inside the shed versus outside the shed (*Water_Inside_Used, Water_Outside_Used*)	Beta (*s* + 1, *n* − *s* + 1) 22 answers of surface water use (*n*), 12 answers use surface water inside shed, 10 answers use surface water outside shed	Scott et al. (11, 13)

11. Water inside chicken sheds is treated	Yes No	Proportion of answers from farmers that treat water inside the chicken sheds (*Water_Inside_Treated*)	Beta (*s* + 1, *n* − *s* + 1) 50 answers of water use inside chicken sheds (*n*), 48 answers of water treated inside chicken sheds	Scott et al. (11, 13)

12. Water outside chicken sheds is treated	Yes No	Proportion of answers from farmers that treat water used outside the shed (*Water_Outside_Treated*)	Beta (*s* + 1, *n* − *s* + 1) 17 answers of water use outside chicken sheds (*n*), 14 answers of water treated outside chicken sheds	Scott et al. (11, 13)

13. Chickens have escaped the shed or range area	Yes No	Proportion of farms that have reported chickens unintentionally outside of the shed or range area (*Escape*)	Beta (*s* + 1, *n* − *s* + 1) 25 farms surveyed (*n*), 21 farms reported chickens escaped shed or range area	Scott et al. (11, 13)

14. Other indirect routes that can lead to LPAI introduction	Yes No	Probability that chickens will be exposed to LPAI virus via other indirect methods; boots, mice/rats, insects and pets combined into one probability (*Indirect*) (Probability of exposure from boots + mice/rats + insects + pets)	*Probability of exposure from boots* (PrBoots) 6/63 answers did not use footbaths AI virus survival on boots is 3/6 days, considered high probability of exposure PrBoots = (6/63) × [Uniform (0.7, 1)] *Probability of exposure from mice/rats* (PrMice) 19/63 answers had mice/rats in sheds 12 mice inoculated (*n*), 0 positive on virus isolation PrMice = (10/25) × [Beta (*s* + 1, *n* − *s* + 1)] *Probability of exposure from insects* (PrInsects) 25/63 answers had insects in sheds 171 insects tested (*n*), 73 positive on virus isolation PrInsects = (25/63) × [Beta (*s* + 1, *n* − *s* + 1)] *Probability of exposure from pets* (PrPets) 13/63 answers allowed pets in sheds on range area AI virus survival on feces is 2/6 days, considered moderate probability of exposure PrBoots = (13/63) × [Uniform (0.3, 0.5)]	Scott et al. (11, 13) Henzler et al. (24) Achenbach and Bowen (19) Nielsen et al. (20) Tiwari et al. (21) Nazir et al. (22)

### Sensitivity Analysis

The Advanced Sensitivity Analysis tool of the program @RISK 7.0 (Palisade Corporation, USA) was used to determine the impact of changes in the input parameters on the model outputs.

The effect of the following inputs on exposure were investigated: (1) proportion of waterfowl on property (*Prop_WF*); (2) proportion of waterfowl on waterbodies (*WB_WF*); (3) proportion of waterfowl in feed storage areas (*F_WF*); (4) proportion of waterfowl on the range (*R_WF*); (5) Farm use of surface water (*Surface_Water_Used*); (6) water inside the chicken shed is treated (*Water_Inside_Treated*); (7) water outside the chicken shed is treated (*Water_Outside_Treated*); (8) escapee chickens from the shed or range (*Escape*); and (9) indirect exposure to the virus (*Indirect*). The influence of the prevalence of LPAI (*Prev_WF)* in waterfowl was also investigated separately.

The values for the input parameters included in the sensitivity analysis were varied from 0 to 1 in thirds (0, 0.3, 0.6, 1), with 1,000 iterations used for each of the values included, while all other input values were fixed to their base value. The model outputs assessed were the overall probability of exposure to LPAI across the three seasons per farm type.

## Results

### Probability of Direct and Indirect Exposure

The probability of a first LPAI exposure to a chicken on a commercial chicken farm being exposed from a wild bird present on the farm at any point in time through the pathways considered in this assessment was estimated to be extremely low for all farm types (Table 6). The assessment estimates a median (5–95%) overall probability of LPAI exposure on commercial free-range layer farms to be 7.5 × 10^−4^ (5.7 × 10^−4^–1.0 × 10^−4^); this being the highest probability among all farm types. The farm type with the lowest estimated overall probability of LPAI exposure was the barn layer farm type (3.0 × 10^−4^; 1.4 × 10^−4^–5.8 × 10^−4^).

**Table 6:** Median (5 and 95 percentiles) probabilities of direct and indirect exposure of a chicken on the commercial chicken farm types (non-free-range meat chicken, free-range meat chicken, cage layer, barn layer, free-range layer) to low pathogenic avian influenza (LPAI) viruses for the first time at any point in time from wild birds in Australia (specifically in the Sydney basin bioregion and South East Queensland).

Exposure and farm type	Median	5%	95%	*F*statistic (degrees of freedom); *p*-value
**Overall probability of exposure (direct and indirect)**				
Non-free-range meat chicken	0.00037	0.00020	0.00064	*F*(4,4995) = 1812.63; <0.0001
Free-range meat chicken	0.00032	0.00018	0.00057	
Cage layer	0.00032	0.00015	0.00063	
Barn layer	0.00030	0.00014	0.00058	
Free-range layer	0.00075	0.00057	0.00010	
**Probability of direct exposure**				
Non-free-range meat chicken	8.68 × 10^−5^	3.153 × 10^−5^	0.00019	*F*(4,4995) = 8927.21; <0.0001
Free-range meat chicken	0.00016	8.45 × 10^−5^	0.00030	
Cage layer	0.00011	3.81 × 10^−5^	0.00025	
Barn layer	8.82 × 10^−5^	3.00 × 10^−5^	0.00022	
Free-range layer	0.00056	0.00043	0.00073	

**Probability of indirect exposure**				
Non-free-range meat chicken	0.00027	0.00014	0.00053	*F*(4,4995) = 235.78; <0.0001
Free-range meat chicken	0.00016	5.72 × 10^−5^	0.00036	
Cage layer	0.00020	7.76 × 10^−5^	0.00047	
Barn layer	0.00019	7.46 × 10^−5^	0.00045	
Free-range layer	0.00017	9.38 × 10^−5^	0.00036	

**Overall probability of exposure (direct and indirect—5 sheds on the property)**				
Non-free-range meat chicken	0.00185	0.001	0.0032	*F*(4,4995) = 1878.45; <0.0001
Free-range meat chicken	0.0016	0.0009	0.00285	
Cage layer	0.0016	0.00075	0.00315	
Barn layer	0.0015	0.0007	0.0029	
Free-range layer	0.00375	0.00285	0.0005	

**Overall probability of exposure (direct and indirect—10 sheds on the property)**				
Non-free-range meat chicken	0.0037	0.002	0.0064	*F*(4,4995) = 1878.45; <0.0001
Free-range meat chicken	0.0032	0.0018	0.0057	
Cage layer	0.0032	0.0015	0.0063	
Barn layer	0.003	0.0014	0.0058	
Free-range layer	0.0075	0.0057	0.001	

When the type of LPAI exposure was considered, direct exposure had the highest probability of causing first exposure to a domestic chicken among free-range farm types, with the lowest being reported for barn layer farms (Table 6). By contrast, the probability of indirect exposure was highest in non-free-range farm types.

To assess the influence of flock size of the farm on the probability of exposure, the overall probability of exposure of each farm type was multiplied by hypothetical numbers of sheds on the property, as each shed can be considered independent in the exposure models. Five and 10 sheds were used, and results are shown in Table 6.

### Estimated Number of Exposures According to Volume of Wild Birds

**Figure 4:**
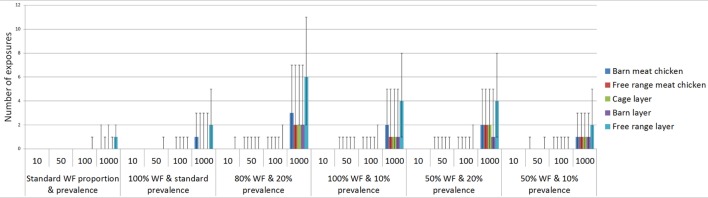
Number of low pathogenic avian influenza (LPAI) virus exposures that would occur given a number of wild birds (*n*) and changes in the overall probability of LPAI exposure (*p*) with changes in the proportion of wild birds on the farm that are waterfowl and the prevalence of LPAI in waterfowl for the commercial poultry farm types (non-free-range meat chicken, free-range meat chicken, cage layer, barn layer, free-range layer) at any point in time out of 100 scenarios (or farms) using binomial distributions; WF, waterfowl.

Results from the binomial distributions are shown in Table 7 and Figure 4. According to the model, a considerable number of wild birds are required for exposure to occur across all farm types. The output distributions indicate that for all farm types, except free-range layer farms, when 1,000 wild birds are present at any point in time around the farm, only on 5 of 100 farms (or scenarios) would one exposure occur, indicating that in 94.9% of farms, exposure would not occur. For free-range layer farms, only 100 wild birds are required to be present to expect a similar exposure output. In instances where 1,000 birds visit at any point in time on free-range layer farms, on 50 out of 100 farms (or scenarios), one LPAI exposure would occur based on the median of one in Table 7.

**Table 7:** Number of low pathogenic avian influenza (LPAI) virus exposures that would occur given a number of wild birds (*n*) and changes in the overall probability of LPAI exposure (*p*) with changes in the proportion of wild birds on the farm that are waterfowl and the prevalence of LPAI in waterfowl for the commercial chicken farm types (non-free-range meat chicken, free-range meat chicken, cage layer, barn layer, free-range layer) at any point in time out of 100 scenarios (or farms) using binomial distributions.

Waterfowl proportion		Standard			100%			80%			100%			50%			50%		
							
Waterfowl LPAI prevalence		Standard			Standard			20%			10%			20%			10%		
						
Farm type	Number of wild birds	Median	5%	95%	Median	5%	95%	Median	5%	95%	Median	5%	95%	Median	5%	95%	Median	5%	95%
Non-free-range meat chicken	10	0	0	0	0	0	0	0	0	0	0	0	0	0	0	0	0	0	0
	50	0	0	0	0	0	0	0	0	1	0	0	1	0	0	1	0	0	1
	100	0	0	0	0	0	1	0	0	1	0	0	1	0	0	1	0	0	1
	1,000	0	0	2	1	0	3	3	0	7	2	0	5	2	0	5	1	0	3

Free-range meat chicken	10	0	0	0	0	0	0	0	0	0	0	0	0	0	0	0	0	0	0
	50	0	0	0	0	0	0	0	0	1	0	0	1	0	0	1	0	0	0
	100	0	0	0	0	0	1	0	0	1	0	0	1	0	0	1	0	0	1
	1,000	0	0	1	1	0	3	2	0	7	1	0	5	2	0	5	1	0	3

Cage layer	10	0	0	0	0	0	0	0	0	0	0	0	0	0	0	0	0	0	0
	50	0	0	0	0	0	0	0	0	1	0	0	1	0	0	1	0	0	0
	100	0	0	0	0	0	1	0	0	1	0	0	1	0	0	1	0	0	1
	1,000	0	0	2	1	0	3	2	0	7	1	0	5	2	0	5	1	0	3

Barn layer	10	0	0	0	0	0	0	0	0	0	0	0	0	0	0	0	0	0	0
	50	0	0	0	0	0	0	0	0	1	0	0	1	0	0	1	0	0	0
	100	0	0	0	0	0	1	0	0	1	0	0	1	0	0	1	0	0	1
	1,000	0	0	1	1	0	3	2	0	7	1	0	5	1	0	5	1	0	3

Free-range layer	10	0	0	0	0	0	0	0	0	1	0	0	0	0	0	0	0	0	0
	50	0	0	0	0	0	1	0	0	1	0	0	1	0	0	1	0	0	1
	100	0	0	1	0	0	1	0	0	2	0	0	2	0	0	2	0	0	1
	1,000	1	0	2	2	0	5	6	2	11	4	1	8	4	1	8	2	0	5

The number of exposures was assessed by changing the proportion of wild birds that are waterfowl and the LPAI prevalence of waterfowl, with some scenarios representing worst-case scenarios (with high proportion of waterfowl present among the wild birds on farm and with elevated LPAI prevalence among the wild birds on farm). Waterfowl may make up a considerable proportion of wild birds on the property during specific events such as drought and, similarly, the prevalence of LPAI in waterfowl may increase with population dynamics, such as an increase in immune-naive juvenile birds. The impact of these scenarios on the number of expected exposures is shown in Table 7. The largest number of exposures is seen when the proportion of waterfowl is increased to 80% and the prevalence increased to 20%. For all farm types, other than free-range layer farms, at least one exposure would occur when 50 wild birds are present at the property, and this would occur on 5 out of 100 farms (or scenarios). Only 10 wild birds are required for an exposure to occur in these circumstances for free-range layer farms.

### Exposure Assessment in the Three Seasons

The estimated probabilities of a chicken on commercial chicken farms being exposed to LPAI virus from wild birds at any point in time during the three seasons previously defined (winter, autumn/spring, and summer) are summarized in Table 8. The results show that the overall probability of exposure differs between the different seasons and the season influence also differs between farm types. While the median overall probability of exposure to LPAI virus is highest in winter for free-range layer farms, this probability is highest in autumn/spring for non-free-range meat chicken, cage layer, and barn layer farm types. No difference between winter and autumn/spring was observed for free-range meat chicken farms. For all farm types, the lowest median overall probability of LPAI virus exposure is in summer.

**Table 8:** Median (5 and 95 percentiles) overall probabilities of exposure (direct and indirect) of a chicken on the commercial chicken farm types (non-free-range meat chicken, free-range meat chicken, cage layer, barn layer, free-range layer) to low pathogenic avian influenza (LPAI) viruses for the first time at any point in time during the three defined seasons; winter (June–August); summer (December–February); and autumn and spring (March–May and September–November); from wild birds in Australia (specifically in the Sydney basin bioregion and South East Queensland).

Farm type	Median	5%	95%	*F*statistic (degrees of freedom); *p*-value
**Winter**				
Non-free-range meat chicken	0.00044	0.00024	0.00079	*F*(4,4995) = 2327.39; <0.0001
Free-range meat chicken	0.00039	0.00022	0.00068	
Cage layer	0.00038	0.00017	0.00077	
Barn layer	0.00035	0.00016	0.00070	
Free-range layer	0.00102	0.00076	0.0014	

**Summer**				
Non-free-range meat chicken	0.00019	0.00010	0.00034	*F*(4,4995) = 403.78; <0.0001
Free-range meat chicken	0.00018	9.06 × 10^−5^	0.00035	
Cage layer	0.00018	8.09 × 10^−5^	0.00036	
Barn layer	0.00017	7.56 × 10^−5^	0.00033	
Free-range layer	0.00030	0.00020	0.00049	

**Autumn/Spring**				
Non-free-range meat chicken	0.00046	0.00026	0.00082	*F*(4,4995) = 1525.98; <0.0001
Free-range meat chicken	0.00039	0.00023	0.00069	
Cage layer	0.00040	0.00018	0.00079	
Barn layer	0.00036	0.00017	0.00072	
Free-range layer	0.00093	0.00069	0.0012	

### Exposure Sensitivity Analysis

**Figure 5:**
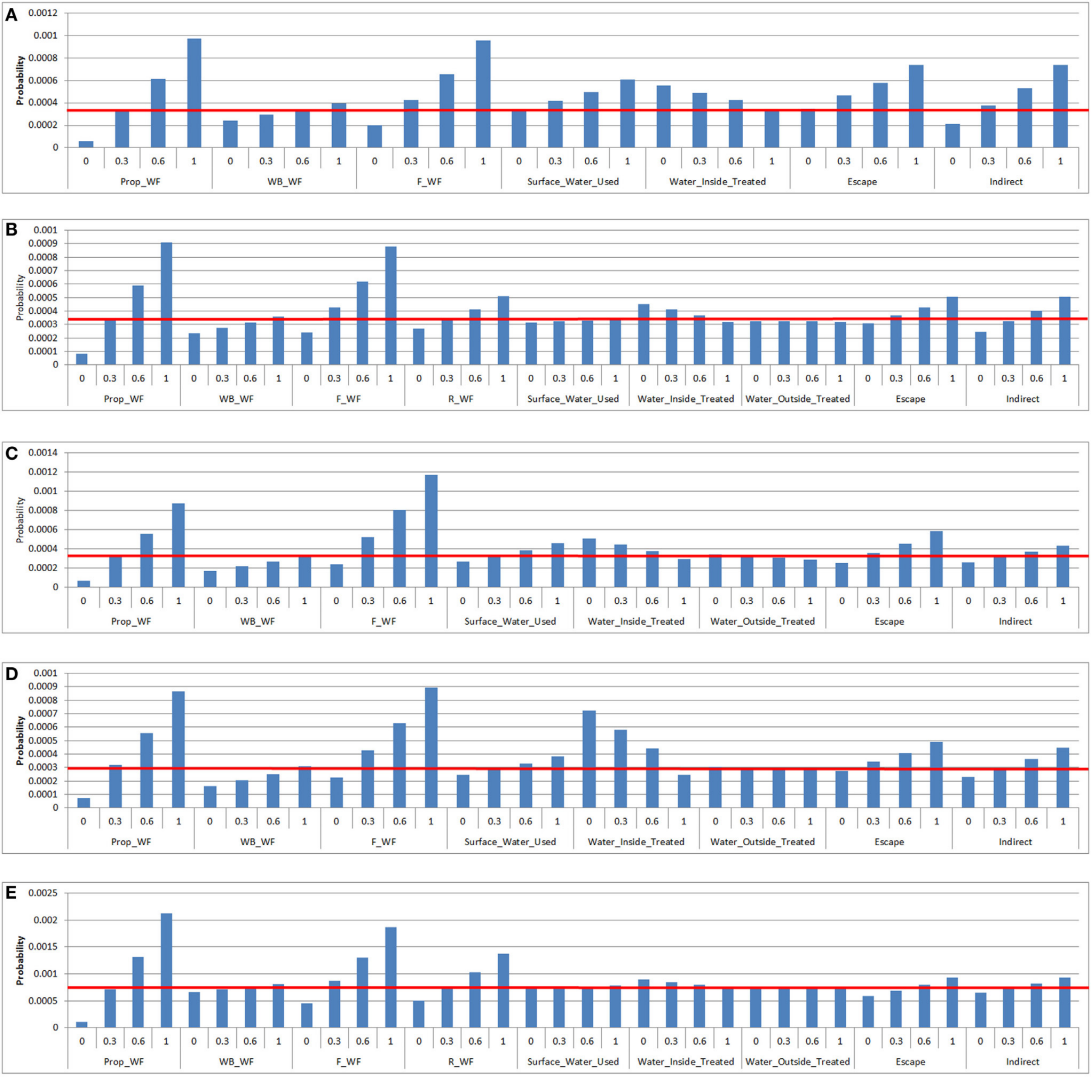
Results of the sensitivity analysis on the exposure assessment depicting the change in probability (*Y*-axis) on the median overall probability of exposure (horizontal line) of a chicken on a commercial chicken farm to low pathogenic avian influenza (LPAI) virus from wild birds in Australia with changes of certain input variables listed in Tables 4 and 5 (*X*-axis). Results were obtained from a simulation of 1,000 iterations using @Risk’s Advanced Sensitivity Analysis. [**(A)** = non-free-range meat chicken; **(B)** = free-range meat chicken; **(C)** = cage layer; **(D)** = barn layer; **(E)** = free-range layer]; Prop_WF, proportion of waterfowl reported on property, WB_WF, proportion of responses that witnessed waterfowl in waterbodies on/near the farm, F_WF, proportion of responses that witnessed waterfowl in feed storage areas, R_WF, proportion of responses that witnessed waterfowl on the range, Surface_Water_Used, proportion of responses that use surface water for the chicken farm, Water_Inside_Treated, proportion of responses that treat water used inside sheds, Water_Outside_Treated, proportion of responses that treat water used outside sheds, Escape, proportion of farms that reported chickens escaping from shed [and from range for **(B)** and **(E)**], Indirect, probability of the occurrence of other indirect methods that can introduce LPAI (boots, mice/rats, insects, farm cats, or dogs).

According to the exposure sensitivity analysis, the most influential parameters were the proportion of waterfowl among wild birds on the property and the probability of waterfowl being on the feed storage areas (Figure 5). When the proportion of waterfowl among wild birds on the property becomes 100% (base value between 0.28 and 0.34 for all farm types), which can occur during circumstances such as drought, there is an approximate 2.8-fold increase in the probability of LPAI exposure for all farm types. A similar increase in the probability of LPAI exposure is obtained when the probability of waterfowl being on feed storage areas is increased to 100%. On free-range farms (Figures 5A,B), waterfowl on the range was the third most influential parameter. When the probability of waterfowl on the range is increased to 100% (base value 0.50 and 0.28 for free-range meat chicken and layer farms, respectively), an approximate 1.7-fold increase in the probability of LPAI exposure occurs. On barn layer farm types, the treatment of water inside the shed is also an important influential parameter. If the probability of water inside sheds being appropriately treated is decreased to 0% (base value 0.92 for barn layer farms), there is an approximate 2.4-fold increase in the probability of LPAI exposure. For other farm types, the impact of water treatment is not as significant. Escapee chickens from the sheds or the range was another influential parameter; if the probability of this occurring is increased to 100% (base value 0.042, 0.042, 0.45, 0.067, and 0.46 for non-free-range meat chicken, free-range meat chicken, cage layer, barn layer and free-range layer farms, respectively), an approximate 1.7-fold increase in the probability of LPAI exposure occurs for all farm types. The other indirect routes parameter (includes boots, mice/rats, insects, farm cats, or dogs) was slightly less influential, with an approximate 1.5-fold increase in the probability of LPAI exposure if the probability of these pathways occurring increases to 100% (base value of 0.28–0.43 for all farm types). The proportion of waterfowl on waterbodies, the use of surface water, and the treatment of water outside of sheds were found to be the least influential parameters in the exposure probability for all farm types.

Sensitivity analysis on the prevalence of LPAI in waterfowl was also conducted separately as this parameter has a profound influence on the probability of LPAI exposure. It was found from the sensitivity analysis there is an approximate threefold to fourfold increase in the probability of LPAI exposure across the farm types when the LPAI prevalence in wild waterfowl is increased to 20%.

## Discussion

This study comparatively estimates the probability of a first exposure of a chicken to LPAI viruses from wild birds present on different types of commercial chicken enterprises in Australia. The probabilities estimated in this study can be considered representative for the Sydney basin region as weather information and the majority of on-farm surveys conducted are specific to this region. In addition, all of the LPAI wild bird prevalence data used in this study was from the Sydney basin region, where most samples were collected from the Lower Hunter region which was considered part of the Sydney basin region in the survey by Scott et al. (11). Generalizing these probabilities to commercial chicken farms in other regions of Australia, non-commercial chicken farms or farms with poultry species other than chickens must be done with caution as differences in farm design and management and biosecurity practices exist as well as differences in weather conditions and LPAI wild bird prevalence in different regions (1, 23, 25). Further research is required to confidently quantify the risk of exposure to commercial chicken farms in other regions, to non-commercial chicken farms and other poultry farms.

### Probability of LPAI Exposure

The probabilities of exposure estimated in this study apply to commercial chicken farms according to the definition implemented in the on-farm survey conducted by Scott et al. (11) as this survey provided data that informed most input parameters. This survey included layer and meat chicken farm which house more than 1,000 or 25,000 chickens, respectively. Thus, the model outputs in this study apply to these flock sizes. There is epidemiological evidence that large flock sizes may be at greater risk of HPAI introduction compared to small flock sizes (26). There is limited information to suggest that this is also true for LPAI introduction, but it is logical to acknowledge that large flock sizes have more animal contacts which may increase the risk of LPAI exposure. This study assessed the influence of flock size on the overall probability by considering the number of sheds on the property and demonstrated that more sheds on a property lead to greater probabilities of exposure.

Overall, the probability of a first exposure to LPAI from wild birds at any point in time is extremely low for all farm types; however, the highest probability of exposure is seen among free-range layer farms, with this probability being over two times higher than for the other farm types. These results are in agreement with a study conducted by Gonzales et al. (27), which reported a rate of introduction of LPAI virus 13 times higher in outdoor layer farms when compared to indoor layer farms in the Netherlands. It has been indicated that the most efficient means of introduction of AI into commercial poultry is through direct contact with infected birds (28). Free-range farms have access to the outdoors where direct exposure to wild birds is more likely to occur compared to indoor farms, and this is in agreement with results presented here. However, during on-farm surveys, it was found that Australian free-range meat chickens are relatively restricted in their access to the outdoors, which is determined by their age and suitable outside weather conditions (11). Most Australian meat chickens are processed when they reach only 50 days, whereas layer chickens are kept in production until around 70–80 weeks (11, 25).

There have been a total of 15 confirmed LPAI cases in Australian poultry since 1976 (29, 30). These cases include LPAI detections of various subtypes, including outbreaks and single bird detections, in Australian poultry. These detections have been a result of passive surveillance (diagnostic submissions), active surveillance (during HPAI outbreaks) and incidental findings not associated with disease. Most have occurred in domestic flocks of ducks, with five incidents in combined chicken and duck farms. In addition, breeder birds were involved in several incidents, with two detections in breeder duck farms, two in breeder chicken farms, and two in mixed breeder and meat duck farms. Four cases occurred in meat poultry farms (two turkey and two duck farms). LPAI has never been detected on a meat chicken farm or on a single-species commercial egg layer enterprise (29). The exposure model considers single-species commercial chicken farms only. Therefore, of all LPAI detections that have occurred in Australia so far, comparisons with the model results can only be made with the two LPAI detections that occurred in breeder chicken farms. Breeder chicken farms are essentially equivalent to barn layer chicken farms and usually have good biosecurity (25). However, the exposure model suggests barn layers have the lowest probability of overall LPAI exposure compared to all farm types. As well as good biosecurity, breeder chicken farms tend to also have close flock health monitoring, as the LPAI detections that occurred were during outbreak investigation related to a drop in production performance (29). It is very likely LPAI detections in Australia are underreported as LPAI infections can be non-clinical, especially in ducks (29). This study found that information on AI virus characteristics and behavior, especially in an Australian context, is extremely scarce.

To best validate these models, routine sampling of Australian commercial chicken farms for LPAI should be conducted. According to the Australian Veterinary Emergency Plan for AI (31), farms with positive detections of H5 or H7 AI virus via cloacal or oropharyngeal swabs must be depopulated and quarantine measures put into place. Given the current depopulation policy, the introduction of financial incentives or encouragement from industry is required to convince farmers to participate in active surveillance sampling. Voluntary participation in routine surveillance as part of a farm accreditation program can also be considered (32). As an alternative to this sampling approach, serological surveys can also be used as occurs in the Netherlands, where all poultry farms were tested for evidence of seroconversion at least once a year, with outdoor layer farms being tested three to four times per year. These data were used to estimate the introduction rates between different farm types (27). Serological sampling has also been performed in Australia but in small, sentinel free-range flocks located near waterfowl habitat and far from commercial chicken enterprises. Results from this sampling showed an extremely low introduction rate; from 2,000 samples collected over 8 years, 0.85% (17) samples tested positive for AI antibodies and 4.35% (87) were uncertain. The number of H5 and H7 subtypes was not determined in the study (33). Although useful, this information cannot be confidently applied to commercial chicken enterprises due to stark differences in the number of birds in a flock, management practices, and farm locations.

### Probability of Direct and Indirect LPAI Exposure

The differences in the probability of direct and indirect exposure between free-range and non-free-range farms are likely due to the definitions of exposures types used in this model. Direct exposure is more likely to occur when chickens have access to the outdoors and, as such, exposure to the virus in non-free-range farms is more likely to occur through indirect pathways. Biosecurity refers to actions to prevent the introduction and spread of infectious agents. In relation to poultry enterprises this refers to practices, such as the use of foot baths, treatment of water, disinfection of equipment between sheds, and vermin control (34). It was found during on-farm surveys that non-free-range meat chicken farms were usually older farms with relatively poorer biosecurity compared to free-range meat chicken farms (12). This relative lack in biosecurity contributed to the highest median probability of indirect exposure occurring in non-free-range meat chicken farms compared to the rest of the farm types. This in combination with the relative restriction to the outdoors in free-range meat chicken farms lead to the higher overall probability of LPAI exposure in non-free-range meat chicken farms compared to free-range meat chicken farms. Biosecurity was also relatively lacking in cage layer farms compared to other farm types, where layer chickens were reported to escape the sheds to the feed storage areas and wild birds reported to be inside sheds (11, 12). This explains the relatively high probability of both direct and indirect LPAI exposure in cage layer farms compared to other farm types.

Another major introduction route implicated for LPAI is the contamination of drinking water for chickens with infective wild bird feces. At least half of all Australian HPAI outbreaks so far are likely to have been associated with the introduction of LPAI *via* contaminated drinking water (4, 35). However, on-farm survey results showed a high level of water treatment across all farm types. The treatment methods identified in the on-farm surveys were deemed adequate to deactivate LPAI, due to the fragile nature and short persistence of AI viruses in the environment (21). Therefore, the use of surface water is not a highly influential parameter, also depicted in the sensitivity analyses, due to the high proportion of water treatment among all farm types. Overall, the treatment of water inside and outside sheds were not found to be significantly influential parameters. In general, it was found that water treatment inside sheds was more influential in the indoor, non-free-range farms compared to free-range farms due to the limited opportunities of direct exposure in indoor farm types.

The exposure sensitivity analysis revealed that the most influential parameters were related to waterfowl presence on the farm; particularly the proportion of waterfowl among wild birds on the property, waterfowl around feed storage areas, and waterfowl on the range. Waterfowl on waterbodies was not a highly influential parameter due to the high proportion of farms that treat surface water, as previously mentioned, and the low probability of aerosol transmission of LPAI from wild waterfowl on waterbodies to commercial chickens (18). However, waterbodies are an attractant for waterfowl and artificial waters, such as dams are used extensively by waterfowl (36) and it is expected that waterfowl on waterbodies in proximity to farms will move to feed storage areas or the range of the farm. To effectively reduce the probability of LPAI exposure to Australian commercial chickens, efforts must be considered to ethically and effectively deter waterfowl from chicken farms. However, farm dams play an important role in water supply and irrigation in Australian agriculture and so the removal of open water sources can be of a great detriment to the farmer (37). In addition, covering open water sources and netting ranges are cost prohibitive (38). Recommendations from a critical review on the deterrence of wild waterfowl from Australian poultry production areas include maintaining optimal grass height, preventing grass going to seed, improving drainage on range areas and around sheds, and prompt cleaning of feed spills around feed storage areas. Other sophisticated recommendations include the development of a 24/7 waterfowl monitoring system on farm and then trialing a range of cost-effective radar-activated on-demand auditory, visual, or physical deterrent systems (38).

### Volume of Wild Birds on the Probability of LPAI Exposure

In addition to the presence of waterfowl in different areas of the farm, the actual number of waterfowl present as well as the prevalence of LPAI in waterfowl are highly influential on the potential number of exposures occurring. The 1994 H7N2 outbreak in Lowood, Queensland is a classic example of both Australian waterfowl movements and the impact of the number of waterfowl in a property. The outbreak occurred during severe drought and a river that constituted one border for the farm as well as a small dam near the entrance of the chicken sheds had attracted a large population of wild birds prior to the subsequent outbreak. LPAI was speculated to be introduced to the flock through contaminated drinking water (4). Currently, there is no available data that accurately estimates the number of wild birds that visit Australian commercial chicken farms over a certain time period. Wildlife camera trapping work conducted by Scott et al. (13) demonstrated an average of 17 wild bird sightings a week. This is very likely an underestimate as the cameras did not capture the whole farm area. However, this data can be extrapolated, and it can be said that approximately 17 wild birds a week is equivalent to approximately 1,000 wild birds a year. Therefore, the number of exposures estimated in this study for 1,000 wild birds present at one point in time could indicate the cumulative expected number of exposures that can occur in one year. Accurate information of wild bird numbers can be obtained from manual wild bird farm surveys or the development of a 24/7 wild bird monitoring system on farm as was stated as a recommendation for wild waterfowl deterrence previously (38).

### The Effects of Season on the Probability of LPAI Exposure

The probability of the first exposure to LPAI virus for a chicken on an Australian commercial chicken farm was found to be lowest in summer for all farm types. The highest probability was estimated to be in winter for chickens on free-range layer farms and autumn/spring for the rest of the farms, except for free-range meat chicken farms which reported no difference between winter and autumn/spring. However, there were minor differences in the probabilities of exposure for all farm types between winter and autumn/spring overall. Among previous HPAI outbreaks in Australia, one occurred in winter (July), four in autumn and spring (May, October, and November), and two in summer (December and January). The three latest outbreaks that occurred in Tamworth (1997), Maitland (2012), and Young (2013) occurred in October or November (4, 39, 40). The mechanisms of mutation from LPAI to HPAI are poorly understood and difficult to predict. In some overseas outbreaks, LPAI viruses have been detected in domestic poultry weeks or months prior to the subsequent HPAI virus outbreaks (41). It could be speculated for the Australian HPAI outbreaks that occurred in summer, when the probability of LPAI exposure is estimated to be lowest compared to the other seasons, which introduction of the virus occurred during spring, the virus then circulated within the flock for months and mutation subsequently occurring in summer (42). On the other hand, Fusaro et al. (43) demonstrated that some H7 LPAI subtypes detected in Italy can mutate quickly in order to adapt to the new host species.

The seasonal variations in the probability of exposure are influenced by the wild bird LPAI prevalence data and the guidelines on outside weather conditions that determine whether or not chickens are provided access to the range. The overall prevalence of LPAI in Australian wild waterfowl at any point in time is approximately 2.5%. Seasonal effects on the prevalence of LPAI in wild birds within NSW do not appear to fluctuate as greatly as in the northern hemisphere (17). There is evidence to suggest that the fluctuation of wild bird LPAI prevalence in Australia is more dependent on rainfall patterns and bird movements, abundance and breeding particularly in Australian waterfowl (44, 45). In the northern hemisphere, there is generally a low prevalence of LPAI in winter, an increase in viral prevalence in summer, followed by a peak in prevalence in autumn (46, 47). This contrasts with NSW data which reveals a high prevalence of LPAI in winter and autumn/spring and a low prevalence in summer (17). In the northern hemisphere, the increased prevalence in summer is thought to be due to the progressive influx of immunonaïve juvenile waterfowl to the population, following breeding in spring (48). In Australia, the breeding seasons and movements of waterfowl are less predictable; many populations are nomadic, which contrasts with the waterfowl populations in the northern hemisphere which are well known for their annual migrations over long distances. Movements and breeding of Australian waterfowl are instead largely determined by the distribution of surface water and rainfall (49, 50). A high prevalence of LPAI may occur during periods of waterfowl congregation, such as during droughts. A particular example that supports this point is the 1994 H7N3 HPAI outbreak that occurred in Queensland, Australia, which took place during a period of severe drought. Water used for the farm was drawn from a river on the periphery of the farm and had attracted a large population of wild birds. This likely greatly increased the probability of LPAI exposure to the farm and lead to the HPAI outbreak (4).

Birds in the families Scolopacidae and Charadriidae (shorebirds and waders) do undergo annual migrations over long distances and visit Australasia (49). In the northern hemisphere, the arrival of migrant birds to the resident population coincides with the peak LPAI prevalence in autumn. Migrating birds may be more susceptible to infection from long distance flights and/or relatively low immune resistance to locally circulating LPAI strains compared to resident birds (48). These shorebirds are more likely to become infected with local Australian LPAI subtypes rather than bring exotic strains of the virus into Australia; the probability of the latter occurring was previously estimated to be extremely low (51).

### Conclusion

There are still many uncertainties related to the mechanisms of the LPAI virus introduction and exposure, particularly in Australian commercial chicken farm settings. However, the results of this study have used the best data available at this time. The results suggest that chickens on commercial free-range layer farms have approximately double the risk of LPAI exposure compared to other farm types. The probability of direct exposure is also more likely in both free-range layer and meat chicken farms compared to the other farm types. Moreover, the probability of LPAI exposure seems to be lower in summer compared to all other seasons and this is influenced by the prevalence of LPAI in wild birds and the weather conditions in which free-range chickens are allowed to go on the range. The proportion of waterfowl on the farm and the presence of waterfowl on the range and feed storage areas are the most influential parameters on the probability of exposure. These results highlight the importance of good biosecurity on farms, providing insight regarding the on-farm actions that can reduce the risk of LPAI exposure such as those related to waterfowl deterrence. In addition, the importance of continuous surveillance of Australian wild bird populations to monitor LPAI prevalence and subtypes is highlighted, as this can help predict future introductions and outbreaks. The need of further research in AI virus properties, particularly in an Australian context is also highlighted.

## Ethics Statement

This study was carried out in accordance with the recommendations of the Human Ethics Committee of the University of Sydney, Australia with written informed consent from all subjects. The protocol was approved by the Human Ethics Committee of the University of Sydney, Australia (ethics reference number: 2015/252).

## Author Contributions

The first author ABS was involved in investigation, methodology, writing of the original draft, and reviewing and editing. J-AT formed initial conceptualization of the study, and was involved in formal analysis, methodology, project administration, supervision of ABS, and reviewing and editing the manuscript. MS was also involved in investigation, methodology, project administration, supervision of ABS, and reviewing and editing the manuscript. BB and KG were also involved in initial conceptualization of the study, formal analysis, methodology, and provided reviewing and editing. BM, AB, and PG were involved in conceptualization and project administration funding/support of the study. PG also provided methodology and supervision. BM provided reviewing and editing. MH-J was heavily involved in formal analysis of the results, conceptualization of the study, methodology, project administration, supervision of ABS, and reviewing and editing the manuscript.

## Conflict of Interest Statement

The authors declare that the research was conducted in the absence of any commercial or financial relationships that could be construed as a potential conflict of interest.

## References

[1] Grillo V Arzey K Hansbro P Hurt A Warner S Bergfeld J Avian influenza in Australia: a summary of 5 years of wild bird surveillance. Aust Vet J (2015) 93(11):387–93. 10.1111/avj.12379 26503532

[2] Swayne D Pantin-Jackwood M Pathobiology of avian influenza virus infections in birds and mammals. In: Swayne D, editor. Avian Influenza. Iowa, USA: Blackwell Publishing (2009). p. 87–122.

[3] Animal Health Australia. Emergency Animal Disease. (2015). Available from: https://www.animalhealthaustralia.com.au/what-we-do/emergency-animal-disease/ (Accessed: March 2, 2016).

[4] Sims LD Turner AJ Avian influenza in Australia. In: Swayne D, editor. Avian Influenza. Iowa, USA: Blackwell Publishing (2009). p. 239–50.

[5] Moloney B Arzey G Wright T Copper K Highly pathogenic avian influenza near Maitland, NSW, in 2012. Proceedings of the Epidemiology Chapter of the Australian and New Zealand College of Veterinary Scientists Scientific Meeting Gold Coast (2013). 47 p.

[6] Roth I Avian influenza outbreak in young – a synopsis. Proceedings of the Australasian Veterinary Poultry Association (AVPA) Scientific Meeting Gold Coast (2014).

[7] Australian Chicken Meat Federation (ACMF) Inc. The Australian Chicken Meat Industry: An Industry in Profile. North Sydney, NSW: Australian Chicken Meat Federation (ACMF) Inc (2011).

[8] Australian Eggs. Annual Report 2016/17. North Sydney, NSW (2017). Available from: https://www.australianeggs.org.au/who-we-are/annual-reports/ (Accessed: January 6, 2018).

[9] New South Wales Department of Primary Industries. NSW Poultry Egg Industry Overview 2015. Orange, NSW: New South Wales Department of Primary Industries (2015).

[10] Singh M Ruhnke I Koning CD Glatz PC Demographics and practices of semi-intesnive free-range farming systems in Australia with an outdoor stocking density of ≤1500 hens/hectare. PLoS One (2017) 12(10):e0187057 10.1371/journal.pone.0187057 29065169 PMC5655439

[11] Scott AB Singh M Toribio JA Hernandez-Jover M Barnes B Glass K Comparisons of management practices and farm design on Australian commercial layer and meat chicken farms: cage, barn and free range. PLoS One (2017) 12(11):e0188505 10.1371/journal.pone.0188505 29166389 PMC5699831

[12] Scott AB Singh M Groves P Hernandez-Jover M Barnes B Glass K Biosecurity practices on Australian commercial layer and meat chicken farms: Performance and perceptions of farmers. PLoS ONE (2018) 13(4):e0195582 10.1371/journal.pone.0195582 29668707 PMC5906091

[13] Scott AB Phalen D Hernandez-Jover M Singh M Groves P Toribio AJ-AL Wildlife presence and interactions with chickens on Australian commercial chicken farms assessed by camera traps. Avian Dis (2017). 10.1637/11761-101917-Reg.1 29620454

[14] World Organisation for Animal Health (OIE). Chapter 2.1. Import Risk Analysis. Terrestrial Animal Health Code. Paris: World Organisation for Animal Health (OIE) (2011). Available from: https://www.oie.int/doc/ged/D10905.PDF (Accessed: July 15, 2015).

[15] Scott AB Toribio J-ALML Singh M Groves P Barnes B Glass K Low- and high-pathogenic avian influenza H5 and H7 spread risk assessment within and between Australian commercial chicken farms. Front Vet Sci (2018) 5:63 10.3389/fvets.2018.00063 29686993 PMC5900437

[16] Martin P Cameron A Greiner M Demonstrating freedom from disease using multiple complex data sources 1: a new methodology based on scenario trees. Prev Vet Med (2007) 79(2-4):71–97. 17224193 10.1016/j.prevetmed.2006.09.008

[17] Hansbro PM Warner S Tracey JP Arzey KE Selleck P O’Riley K Surveillance and analysis of avian influenza viruses, Australia. Emerg Infect Dis (2010) 16(12):1896–904. 10.3201/eid1612.100776 21122219 PMC3294589

[18] Jonges M Leuken JV Wouters I Koch G Meijer A Koopmans M Wind-mediated spread of low pathogenic avian influenza virus into the environment during outbreaks at commercial poultry farms. PLoS One (2015) 10(5):e0125401 10.1371/journal.pone.0125401 25946115 PMC4422664

[19] Achenbach JE Bowen RA. Transmission of avian influenza A viruses among species in an artificial barnyard. PLoS One (2011) 6(3):e17643. 10.1371/journal.pone.0017643 21483843 PMC3069003

[20] Nielsen AA Sovgard H Stockmarr A Handberg KJ Jorgensen PH. Persistence of low-pathogenic avian influenza H5N7 and H7N1 subtypes in house flies (Diptera: Muscidae). J Med Entomol (2011) 48(3):608–14. 10.1603/ME11017 21661322 PMC7107468

[21] Tiwari A Patnayak DP Chander Y Parsad M Goyal SM. Survival of two avian respiratory viruses on porous and nonporous surfaces. Avian Dis (2006) 50(2):284–7. 10.1637/7453-101205R.1 16863083

[22] Nazir J Haumacher R Ike AC Marschang RE. Persistence of avian influenza viruses in lake sediment, duck feces, and duck meat. Appl Environ Microbiol (2011) 77(14):4981–5. 10.1128/AEM.00415-11 21622783 PMC3147373

[23] Bureau of Meterology. Weather Station Directory. (2016). Available from: http://www.bom.gov.au/climate/data/stations/ (Accessed: February 4, 2016).

[24] Henzler D Kradel D Davison S Ziegler A Singletary D DeBok P Epidemiology, production losses, and control measures associated with an outbreak of avian influenza subtype H7N2 in Pennsylvania (1996–98). Avian Dis (2003) 47:1022–36. 10.1637/0005-2086-47.s3.1022 14575105

[25] Scott P Turner A Bibby S Chamings A In: Department of Agriculture Fisheries and Forestry, editor. Structure and Dynamics of Australia’s Commercial Poultry and Ratite Industries. Moonee Ponds, VIC: Scolexia Animal and Avian Health Consultancy (2009).

[26] Thomas M Bouma A Ekker H Fonken A Stegeman J Nielen M. Risk factors for the introduction of high pathogenicity avian influenza virus into poultry farms during the epidemic in the Netherlands in 2003. Prev Vet Med (2005) 69:1–11. 10.1016/j.prevetmed.2004.12.001 15899292

[27] Gonzales JL Stegeman JA Koch G Wit SJD Elbers AR Rate of introduction of a low pathogenic avian influenza virus infection in different poultry production sectors in the Netherlands. Influenza Other Respi Viruses (2012) 7(1):6–10. 10.1111/j.1750-2659.2012.00348.x PMC5780726 22376126

[28] Swayne D Epidemiology of avian influenza in agricultural and other man-made systems. In: Swayne D, editor. Avian Influenza. Iowa, USA: Blackwell Publishing (2009). p. 59–86.

[29] Arzey G Low pathogenic avian influenza in Australia and implications. Proceedings of the Australasian Veterinary Poultry Association (AVPA) Scientific Meeting Sydney (2013).

[30] Australian Chief Veterinary Office. Detection of Low Pathogenic (LP) and Highly Pathogenic (HP) Avian Influenza Virus (AIV) in Flocks of Layers, Breeders and Meat Birds in Australia. Rural and Regional Affairs and Transport Legislation Committee (2015). Available from: https://www.aph.gov.au/~/media/Committees/rrat_ctte/estimates/bud_1516/ag/answers/QoN62-64.pdf (Accessed: January 10, 2018).

[31] Animal Health Australia. Disease Strategy: Avian Influenza (Version 3.4). Australian Veterinary Emergency Plan (AUSVETPLAN). 3rd ed Canberra, ACT: Primary Industries Ministerial Council (2011).

[32] United States Department of Agriculture. National Poultry Improvement Plan. Georgia, USA: Animal and Plant Health Inspection Service (2017). Available from: www.poultryimprovement.org/ (Accessed: January 6, 2018).

[33] East I Ainsworth C Warner S Dunowska M Azuolas J. Seroconversion to avian influenza virus in free-range chickens in the Riverland region of Victoria. Aust Vet J (2010) 88(8):290–3. 10.1111/j.1751-0813.2010.00601.x 20633162

[34] Department of Agriculture Fisheries and Forestry. National Farm Biosecurity Manual Poultry Production. Canberra, ACT: Commonwealth of Australia (2009).

[35] Alexander D Capua I Avian influenza in poultry. Worlds Poult Sci J (2008) 24:513–32. 10.1017/S0043933908000184

[36] Read J A strategy for minimizing waterfowl deaths on toxic waterbodies. J Appl Ecol (1999) 36:345–50. 10.1046/j.1365-2664.1999.00407.x

[37] Tingey-Holyoak JL Water sharing risk in agriculture: perceptions of farm dam management accountability in Australia. Agric Water Manag (2014) 145:123 10.1016/j.agwat.2014.02.011

[38] Atzeni M Fielder D Thomson B Deterrence of wild waterfowl from poultry production areas: a critical review of current techniques and literature. Rural Industries Research and Development Corporation (RIRDC). Wagga Wagga: Australian Government (2016).

[39] New South Wales Government. Highly Pathogenic Avian Influenza (H7N7) Maitland- Control and Eradication. (2012). Available from: http://archive.dpi.nsw.gov.au/content/media-releases/2012/avian-influenza-hunter (Accessed: April 5, 2015).

[40] Department of Agriculture and Water Resources. High Pathogenic Avian Influenza – Young, NSW. (2013). Available from: http://www.agriculture.gov.au/about/media-centre/communiques/high-pathogenic-avian-influenza-a-young-nsw (Accessed: March 2, 2016).

[41] Stech J Mettenleiter TC Virulence determinants of high-pathogenic avian influenza viruses in gallinaceous poultry. Future Virol (2013) 8(5):459–68. 10.2217/fvl.13.27

[42] Richard M Fouchier R Monne I Kuiken T Mechanisms and risk factors for mutation from low to highly pathogenic avian influenza virus. Eur Food Saf Auth (2017) 14(10):EN–1287. 10.2903/sp.efsa.2017.EN-1287

[43] Fusaro A Tassoni L Hughes J Milani A Salviato A Schivo A Evolutionary trajectories of two distinct avian influenza epidemics: parallelisms and divergences. Infect Genet Evol (2015) 34:457–66. 10.1016/j.meegid.2015.05.020 26003682

[44] Tracey JP Risk-based surveillance of avian influenza in Australia’s wild birds. Wildl Res (2010) 37(2):134–44. 10.1071/WR09152

[45] Ferenczi M Beckmann C Warner S Loyn R O’Riley K Wang X Avian influenza infection dynamics under variable climatic conditions, viral prevalence is rainfall driven in waterfowl from temperate, south-east Australia. Vet Res (2016) 47:23. 10.1186/s13567-016-0308-2 26852115 PMC4744453

[46] Causey D Edwards SV Ecology of avian influenza virus in birds. J Infect Dis (2008) 197:29–33. 10.1086/524991 PMC7110149 18269325

[47] Vandegrift KJ Sokolow SH Daszak P Kilpatrick AM. Ecology of avian influenza viruses in a changing world. Ann N Y Acad Sci (2010) 1195:113–28. 10.1111/j.1749-6632.2010.05451.x 20536820 PMC2981064

[48] van-Dijk JG Hoye BJ Verhagen JH Nolet BA Fouchier RA Klaassen M. Juveniles and migrants as drivers for seasonal epizootics of avian influenza virus. J Anim Ecol (2014) 83:266–75. 10.1111/1365-2656.12131 24033258 PMC3869896

[49] Tracey JP Woods R Roshier D West P Saunders GR The role of wild birds in the transmission of avian influenza for Australia: an ecological perspective. Emu (2004) 104:109–24. 10.1071/MU04017

[50] Cooper RM McAllan IA Curtis BR An Atlas of the Birds of NSW and the ACT. Gordon, NSW: Minipublishing (2014).

[51] East IJ Hamilton S Garner G. Identifying areas of Australia at risk of H5N1 avian influenza infection from exposure to migratory birds: a spatial analysis. Geospat Health (2008) 2(2):203–13. 10.4081/gh.2008.244 18686269

