# Supplementary material for: Low Pathogenic Avian Influenza Exposure Risk Assessment in Australian Commercial Chicken Farms
Source: Front Vet Sci. 2018 Apr 26;5:68. doi: 10.3389/fvets.2018.00068 (PMC5932326; doi:10.3389/fvets.2018.00068)
Supplement: Supplementary file 1 [file Data_Sheet_1.docx]

*Type of wild bird on farm property.* This category node estimates the probability that waterfowl, shorebirds or other bird types are present on the farm (*Prop_WF, Prop_SH, Prop_O*). This node accounts for the different LPAI prevalence among these three types of wild birds (Grillo et al., 2015). Waterfowl and shorebirds are birds in the taxonomic orders Anseriformes and Charadriiformes, respectively. These orders constitute the major natural reservoir of influenza A viruses of all subtype combinations (Olsen et al., 2006). Specifically, birds of the family Anatidae, order Anseriformes, constitute the greatest number of virus detections out of all wild bird species (Alexander and Capua, 2008). The branches for this node are the three bird types and all nodes that follow these three branches are identical. Information used to estimate this input parameter was obtained from the survey on commercial layer and meat chicken farms by Scott et al. (2017b; a). In this study, farmers were asked if any of these three bird groups were seen inside chicken sheds, feed storage areas, waterbodies or on the range. For each farm type, answers for each of the three bird groups in all locations were combined. Results from the survey indicate that there are significant differences on the proportion of farms with some of the wild bird types considered in the study, with shorebirds being more prevalent (p = 0.031) in free-range layer farms than in other farm types. A grand total of all answers was then obtained. Beta distributions for each bird type were created using the grand total of all answers as (n) and the total number of answers for each bird group as (s); values are listed in Tables 1 to 5. The result was three Beta distributions of bird type for each farm type. The proportions represented by each of these distributions for each farm type were then used as input parameters in the model.

*Prevalence of LPAI in wild birds.* This probability node estimates the probability that waterfowl, shorebirds or other bird types on the farm during certain times of the year are infected and excreting LPAI virus (*Prev_WildBird_Season; Prev_WF_Winter, Prev_WF_Summer, Prev_WF_AuSp, Prev_SH_Winter, Prev_SH_Summer, Prev_SH_AuSP, Prev_O_Winter, Prev_O_Summer, Prev_O_AuSp*). The branches for this node are yes and no i.e. the respective bird type is or is not infected and excreting LPAI virus respectively. If no, then the model pathway ends at no exposure. If yes, the model pathway continues to nodes relating to the locations of the wild birds, initially at the shed level. Information used to estimate this input parameter was obtained from national surveillance data collected from two sources. One source was from unpublished data from NSW partners for the period from 2007 to 2015 of the National Avian Influenza Wild Bird (NAIWB) Surveillance Program. Positive influenza A detections and total samples collected from waterfowl and shorebirds were used to create Beta distributions for waterfowl and shorebird prevalence for each season; winter (June to August), summer (December to February) and autumn and spring as one season (March to May and September to November). These values can be obtained by contacting the corresponding author to seek permission from the NSW partners of the NAIWB Surveillance Program. The second source was from the study by Hansbro et al. (2010), where a total of 14,782 samples from fresh faeces or cloacal swabs were collected around Newcastle and Orange in NSW from 2005 to 2008 and of 1,552 other bird types, one positive sample was obtained. A Beta distribution was performed for the other bird type using these values the distribution was used for all seasons (Beta(2;1552)).

Only viruses of H5 and H7 subtypes have been shown to mutate to HPAI (Alexander, 2007). Information from the NSW partners of the NAIWB Surveillance Program was also used to calculate the prevalence of H5 and H7 virus subtypes in wild birds by dividing the number of H5 and H7 virus subtypes detected by the total number of influenza A virus detections in order to obtain a proportion for each year. This proportion was multiplied by all Beta distributions created for each wild bird type in each season. The values obtained from these multiplications were then used as the input parameters for this node. These values represented the prevalence of H5 and H7 AI virus subtypes in each wild bird type in each season.

*Respective wild bird type reported inside chicken sheds.* This category node estimates the probability that waterfowl, shorebirds or other bird types go inside chicken sheds (*Sheds_Wildbird; Sheds_WF, Sheds_SH, Sheds_O*). Information used to estimate this input parameter was obtained from the survey on commercial layer and meat chicken farms by Scott et al. (2017b; a). The branches for this node are yes and no i.e. wild birds are or are not present inside chicken sheds respectively. If yes, the model pathway ends at ‘exposure’ because it is assumed that if LPAI infected wild birds enter a chicken shed, then the chickens will be exposed to the virus. This type of exposure is classed as direct exposure because chickens can come into direct contact with wild bird faeces and have physical contact with wild birds inside sheds. If no, the model pathway continues to other locations that wild birds are present if not in the chicken sheds. For each farm type, farmers were asked whether or not they witnessed wild birds inside the chicken sheds. No waterfowl or shorebirds were reported inside chicken sheds for all farm types. Other bird types were reported; commonly small birds such as sparrows or finches. As the data suggested the probability of waterfowl and shorebirds being present inside chicken sheds is zero or close to zero, a Pert distribution with a minimum and most likely of zero and a maximum of 0.05 was used for these bird types. For other bird types, Beta distributions were created using the number of farms that had reported wild birds inside sheds and the total number of farms for each farm type; the values used are listed in Tables 1 to 5.

*Respective wild bird type reported in other locations on farm.* This category node estimates the probability that waterfowl, shorebirds or other bird types are present in other locations on the farm; namely waterbodies, feed storage areas or the range (*WB_Wildbird; WB_WF, WB_SH, WB_O; F_Wildbird; F_WF, F_SH, F_O; R_Wildbird; R_WF, R_SH, R_O*). The branches for this node are the three locations. Each branch leads to a unique pathway and for free range farms, all three branches are used. For non-free range farms, only the branches waterbodies and feed storage areas are used since birds do not have a range area in these farm types. Information used to estimate this input parameter was obtained from the survey on commercial layer and meat chicken farms by Scott et al. (2017b; a). Farmers were asked if any of the three bird groups were seen in waterbodies, feed storage areas or on the range. Similar to the first node, answers for each of the three bird groups in each location were combined. The answers were incorporated into the model using Beta distributions; with values listed in Tables 1 to 5. The proportions represented by each of these distributions for each farm type were then used as input parameters in the model.

*Suitable weather conditions for range access.* This probability node estimates the probability that weather conditions outside are suitable for chickens to be allowed out onto the range (*Range_Season; Range_Winter, Range_Summer, Range_AuSp)*. The branches for this node are yes and no i.e. the weather is or is not suitable for chickens to be let outside, respectively. Information used to estimate this input parameter was obtained from the Australian Bureau of Meterology (BOM, 2016) and the survey on commercial layer and meat chicken farms by Scott et al. (2017b). During the survey, free range farmers were asked about weather conditions when range access restricted. In the Sydney basin region, the specific conditions under which range access was permitted for free range meat chicken farms were dry conditions, between 17 and 28^o^C with no severe weather present e.g. strong wind and lightning. This finding that led to the selection of the three seasons; winter (June to August), summer (December to February) and autumn and spring as one season (March to May and September to November). It was realised that the seasons when bird access to the range is significantly restricted are winter and summer, as temperatures are commonly below and above 17 and 28^o^C, respectively. Weather conditions are similar between autumn and spring and therefore they were combined as one season. Free range meat chicken farms in south east Queensland would allow birds outside depending on their own judgment, not based on specific guidelines. Therefore it was assumed the guidelines used in the Sydney basin region would apply to all farms.

Data was received from BOM (2016) which listed hourly temperature and precipitation recordings from 2010 to 2016 from two weather stations in the Sydney basin region; Cooranbong Lake Macquarie (station number 61412) and Horsley Park Equestrian Centre (station number 67119). Two stations were used to gain an average of north and south conditions of the Sydney basin region, where Cooranbong Lake Macquarie and Horsley Park Equestrian Centre are located relatively north and south of the Sydney basin region respectively. All data used was an average of the two weather stations. The data was divided into the three seasons and the total number of recorded hours in each season over the years was obtained. An average total number of recorded hours were 13248, 13666 and 26352 for winter, summer and autumn/spring respectively. For winter, the total average number of hours in which the temperature was greater than 17^o^C was obtained at 1555 hours, and these hours were very likely to be in the day time. For summer, the total average number of hours between 7am to 7pm where the temperature was below 28^o^C was obtained at 6231.5 hours. This time consideration was applied due to the likely possibility that temperatures could be below 28^o^C but at night time, when chickens would not be allowed outside. For autumn and spring, the total average number of hours where the temperature was between 17 and 28^o^C between 7am to 7pm was obtained at 9338.5 hours. Beta distributions on these suitable temperatures for each season were then created; winter (Beta(1556, 11694)), summer (Beta(6232.5, 7435.5)), autumn/spring (Beta(9339.5, 17014.5)).

BOM (2016) defines a wet day as a day greater than 1mm of rain. As range access for chickens is determined by the hour, the total average number of hours for each season where the precipitation was greater than 1mm of rain was obtained at 1755, 8098.5 and 3960.5 hours for winter, summer and autumn/spring respectively. Beta distributions using this precipitation data for each season were then created; winter (Beta(1756, 11494)), summer (Beta(8099.5, 5568.5)), autumn/spring (Beta(3961.5, 22392.5)). A list of severe weather events from 2010 to 2016 in NSW was also obtained from BOM (2016). The list provided the date, longitude and latitude of each severe weather event. Using the longitudes and latitudes, only events that occurred within the Sydney basin region were obtained. Using the dates, the season in which the event occurred was also obtained. A total of 114 severe weather events from 2010 to 2016 were obtained in the Sydney basin region. Of these, one occurred in winter, 64 occurred in summer and 49 occurred in autumn/spring. Beta distributions using this severe weather data for each season were then created; winter (Beta(2, 113)), summer (Beta(65, 50)), autumn/spring (Beta(50, 66)). As these Beta distributions for precipitation and severe weather define unsuitable weather conditions for range access, one minus these Beta distributions was performed to define suitable weather conditions.

The Beta distributions created for each season were added together i.e. the Beta distributions for suitable temperature, suitable precipitation and no severe weather for winter were all added together, and the same was performed for summer and autumn/spring. After each Beta distribution for each season was summed together, a total sum of all these Beta distributions was obtained. The summed Beta distribution for each season was then divided by the total sum of all Beta distributions. This was performed to obtain a proportion for each season and to ensure the final Beta distributions for each season summed to one. It was these final proportions that were used as the input parameters for the node for free range meat chicken farms.

Survey results by Scott et al. (2017b; a) indicated that suitable weather conditions for range access were variably perceived amongst free range layer farmers. There were 33 answers from free range layer farms of weather conditions that would limit range access; 1 answer of too hot, 10 answers of wet conditions, 22 answers of severe weather and 2 answers of no restrictions regardless of weather conditions. Beta distributions of each of these responses were created. As performed for free range meat chicken farms, data from BOM (2016) was used to create Beta distributions of precipitation hours greater than 1mm of rain and severe weather events for the three seasons. Responses from farmers by Scott et al. (2017b; a) indicated free range layer farms would allow range access at higher temperatures than free range meat chicken farms. A temperature of 35^o^C was agreed as the maximum temperature threshold. Average data from the two weather stations from BOM (2016) revealed 0, 54 and 137 hours above 35^o^C in winter, autumn/spring and summer respectively. Beta distributions were performed on these. The Beta distributions of each weather condition in each season were multiplied by the Beta distribution of the corresponding response from the survey results. The result were three weather conditions for each season; severe weather, heat and wet conditions, that took account of farmer responses. The three conditions for each season were then added together and this resulted in a probability that conditions were not suitable for each season. One minus these values gave the final probabilities that the conditions would be suitable for range access. These values were used as the input parameters for the node for free range layer farms.

*Suitable age for range access*. This probability node estimates the probability that the chickens are a suitable age for range access (*Age*). Information used to estimate this input parameter was obtained from the survey on commercial layer and meat chicken farms by Scott et al. (2017b; a). The branches for this node are yes and no i.e. chickens are or are not a suitable age for range access. If age is not suitable, then the pathway ends at no exposure. If age is suitable, the pathway continues to the next node; whether or not birds actually use the range. The survey by Scott et al. (2017b; a) revealed an average age that birds were allowed outside of 21 days and 22.94 weeks for free range meat chicken and layer farms respectively. The average age of flock depopulation was 43.83 days and 87.32 weeks for free range meat chicken and layer farms respectively. These values are also listed in Tables 2 and 5. These proportions were added into the model using Beta distributions to account for uncertainty around these estimates; free range meat chicken (Beta(22, 23.83)), free range layer (Beta(23.94, 65.38)).

*Birds actually go onto the range.* This probability node estimates the probability that chickens in a shed actually go outside and use the range (*Use_Range*). Information used to estimate this input parameter was obtained from the survey on commercial layer and meat chicken farms by Scott et al. (2017b; a). The branches for this node are yes and no i.e. chickens do or do not use the range area. If birds do not use the range area, the pathway ends at no exposure through this pathway. If yes, the pathway ends at exposure, where direct exposure is assumed in this pathway. In the survey by Scott et al. (2017b; a), free range farmers were asked to estimate the proportion of chickens in a shed that actually go outside and use the range in favourable conditions. Beta distributions of each individual response were created for each farm type; therefore 15 and 25 Beta distributions were created for free range meat chicken and layer farms respectively. An average of all the Beta distributions per farm type was created. These averages were used as the final input parameter for the node i.e. the average of the 15 Beta distributions was used for the free range meat chicken model and the average of the 25 Beta distributions was used for the free range layer model. Beta distributions were used to account for uncertainty around these estimates.

*Aerial transmission of LPAI from wild birds on waterbodies.* This probability node estimates the probability that aerial transmission of LPAI will occur from wild birds present on waterbodies on or near the farm (*Aerosol_WB*). Information used to estimate this input parameter was obtained from Jonges et al. (2015). The branches for this node are yes and no i.e. chickens will or will not be exposed to aerosol LPAI. If yes, the pathway ends at exposure where indirect exposure is assumed in this pathway. If no, the pathway continues to the next node; whether or not surface water is used for the chicken farm. The study by Jonges et al. (2015) involved the collection of air samples at various areas surrounding LPAI infected turkey, chicken and wild swan holdings. All procedures were performed in triplicate. Samples at four distances under 100m at different bearings from 83 LPAI H5N2 infected swans were collected; the distances were 4m, 20m, 20m, and 98m. No virus was detected in any of the samples, suggesting aerial transmission of LPAI from wild birds is a rare event. A Beta distribution was created (Beta(Beta(1,13)) and used in the model to account for uncertainty around this estimate. This Beta distribution was used as the final input parameter for this node for all farm types.

*Surface water is used for chickens*. This node follows from the aerial transmission node, which considers that wild birds are present on waterbodies on or near the property (*Surface_Water_Used*). It is reported that the consumption of LPAI contaminated water by commercial chickens is a common transmission route in which commercial chickens can be exposed to the virus (Swayne, 2008). This node estimates the probability that farms of each farm type use surface water for the chicken farm. Information used to estimate this input parameter was obtained from the survey by Scott et al. (2017b; a). The branches for this node are yes and no i.e. surface water is or is not used for the chicken farm. If no, the pathway ends at no exposure through that pathway. If yes, the pathway continues to the next node; methods in which the surface water is used for. The exception is for non-free range meat chicken farms where the yes pathway continues to the node of water treatment inside the shed. This is because Scott et al. (2017b; a) found that no non-free range meat chicken farms use water outside the shed for the chickens, and so the node of methods in which surface water is used for was omitted. The exposure diagram for non-free range meat chicken farms is depicted in Figure 2. The survey by Scott et al. (2017b; a) asked farmers what the sources of water for the chicken farm were. Some farms used multiple water sources. Surface water was any exposed water such as a farm dam or river. Beta distributions were created for the answers for each farm type, using the total number of farms surveyed for each farm type as (n). The values are listed in Tables 1 to 5. These were used as the final input parameters for this node.

*Locations surface water is used for.* This node estimates the probability that surface water is used inside or outside chicken sheds (­*Water_Inside_Used; Water_Outside_Used*). Each branch follows a unique pathway, both commencing at whether or not water inside or outside the shed is treated. Information used to estimate this input parameter was obtained from the cross-section study by Scott et al. (2017b; a). This node was omitted for non-free range meat chicken farms as it was found no farms used water outside the shed for the chickens. The pathways for this farm type are depicted in Figure 2. Of those farmers that use surface water, they were asked what they used the surface water for. The use of water for foggers, drinkers and cooling pads were classed as inside the shed. The use of sprinklers on the roof for cooling purposes and irrigation of the range were classed as outside the shed. Answers were obtained for each farm type. Beta distributions were created using the total answers inside the shed, outside the shed and the grand total for both inside and outside the shed; values are listed in Tables 2 to 5. This was done per farm type. The proportions represented by each of these distributions for each farm type were then used as input parameters in the model.

*Water inside chicken sheds is treated.* This node estimates the probability that water used inside chicken sheds is treated (*Water_Inside_Treated*). The branches are yes or no i.e. water is or is not treated inside chicken sheds. If no, the pathway ends at exposure and is assumed as indirect exposure in this pathway. If yes, then the pathway ends at no exposure through this pathway. It is assumed that any water treatment performed is effective in inactivating the virus since the AI virus is a relatively weak virus (Swayne, 2008). Information used to estimate this input parameter was obtained from the cross-section study by Scott et al. (2017b; a). The total number of farms that use drinkers, cooling pads and foggers inside the shed was obtained and this was summed together as the total number of answers. The total number of farms that treat drinkers, cooling pads and foggers was also obtained and this was summed together as the total number of ‘yes’ to treatment answers. A Beta distribution was performed per farm type, where the total number of answers was (n) and the total number of ‘yes’ to treatment answers was (s). Values are listed in Tables 1 to 5.

*Water outside chicken sheds is treated*. The calculation of this node is similar to the node of treated water inside the shed and estimates the probability that water outside the shed is treated (*Water_Outside_Treated*). Information used was from the survey by Scott et al. (2017b; a). The total number of farms that use sprinklers and irrigation of range was obtained and this was summed together as the total number of answers. The total number of farms that treat sprinklers and irrigation of range was summed together as the total number of ‘yes’ to treatment answers. A Beta distribution was performed per farm type, where the total number of answers was (n) and the total number of ‘yes’ to treatment answers was (s). Values are listed in Tables 2 to 5. This node was omitted in non-free range meat chicken farms as mentioned previously. The branches for this node are yes or no i.e. water is or is not treated outside chicken sheds. If yes, the pathway ends at no exposure through this pathway. If no, the pathway differs between free range and non-free range farms and is depicted on Figures 3 and 1 respectively. In order for non-free range layer farms to be exposed to water outside of the shed, they must escape the shed. Therefore the next node after the yes branch is escapee chickens from the shed. Free range farms have relatively frequent access to the outdoors but their exposure to the virus is dependent on whether or not the chickens go outside. Therefore the pathway that follows the yes branch in this case is the same pathway after the branch of wild birds on the range; considering weather conditions, age and range use. The difference is that exposure is assumed to be indirect in this pathway as exposure to the virus will be through water.

*Chickens escape shed or range area.* This node follows the branch that wild birds are present in the feed storage area and estimates the probability that chickens escape the shed or range area (*Escape*). Information used to estimate this node was derived from the survey by Scott et al. (2017b; a). The branches for this node are yes or no i.e. chickens do or do not escape the shed or range area respectively. If yes, the pathway ends at exposure which is assumed to be direct exposure. As found by Scott et al. (2017b; a), feed storage areas are located directly adjacent to the chicken sheds, mainly in the form of silos. Wild birds present in feed storage areas will also be in the vicinity of the immediate outside area of the chicken shed. If chickens escape they will enter these immediate outside areas. Wild bird presence in these areas leads to direct exposure as defined by physical contact or direct contact between a commercial chicken with wild bird faeces. If the branch is no, the pathway continues to the next node; indirect introductions via fomites or vectors. Farmers were asked whether or not they witnessed chickens escaping the shed or range area. Beta distributions per farm type were then created using the total number of answers as (n) and the total number of ‘yes’ answers as (s). Values are listed in Tables 1 and 5.

*Other indirect routes.* This node estimates the probability that indirect introduction of LPAI virus to commercial chickens occurs through fomites or vectors (*Indirect*). This node considers wild birds are present in feed storage areas i.e. areas directly adjacent to the shed. Indirect introduction via fomites or vectors assumes wild bird faeces is picked up from these areas via fomites or vectors and carried inside the shed. Fomites or vectors considered in this node were boots, mice/rats, insects and farm dogs and cats. This node uses combined information from the survey by Scott et al. (2017b; a) and also on scientific literature reporting LPAI virus survival and detection in other organisms (Henzler et al., 2003; Tiwari et al., 2006; Achenbach and Bowen, 2011; Nazir et al., 2011; Nielsen et al., 2011). Scott et al. (2017b; a) visited farms and recorded the use of foot baths; farmer observation of mice/rats and/or insects inside sheds; and whether farm cats or dogs were allowed on the shed and/or on the range area. For each farm type, answers of no foot baths, presence of mice/rats, presence of insects and access of farm cats or dogs into range and shed were recorded. Values are listed in Tables 1 to 5. The answers for each fomite were divided by the total number of answers to gain a proportion for that fomite.

Scientific literature indicated that it is a rare event for mice and rats to be positive for AI virus after inoculation and concluded these species likely do not play a significant role in transmission of the virus to other species. Rats were inoculated with LPAI virus and zero of 12 rodents were positive upon virus isolation in one study (Achenbach and Bowen, 2011). A Beta distribution on these results was created, where 12 was the total number of rodents (n) and zero was the total number of positive virus isolations (s). In contrast to these results, scientific literature indicated that flies are a potential carrier of AI virus. Nielsen et al. (2011) conducted an experiment in which flies were fed H7N1 and H5N7 LPAI of three different two-fold virus dilutions. Groups of flies fed the same subtype and dilutions had their contents pooled. A total of 171 fly pools were examined, and of those 73 were positive for the virus (Nielsen et al., 2011). A Beta distribution of this result was created using 171 as (n) and 73 as (s).

The survival of LPAI virus subtypes H4N6, H5N1 and H6N8 in duck faeces, lake sediment and duck meat was examined by Nazir et al. (2011) at four temperatures; 30, 20, 10 and 0^o^C. The study determined the number of days required for each subtype to lose 90% of virus infectivity in the different substances. Of interest to this model were the results in duck faeces at 30 and 20^o^C as these temperatures are typical of Australian conditions and duck faeces can potentially be carried by fomites or vectors. The virus survived the longest in lake sediment followed by duck faeces. The average number of days of survival for the three subtypes in duck faeces and lake sediment in 30 and 20^o^C was 3.5 and 11.83 days respectively. Assuming 11.83 days is roughly the maximum number of days of survival of LPAI in 20 and 30^o^C temperatures, the survival time in duck faeces is approximately 30% of the virus’s potential survival time at these temperatures. A uniform distribution assuming a moderate probability of survival in duck faeces was therefore created (Uniform(0.3,0.7)). Similarly, the survival of LPAI virus subtype H13N7 on gumboots as well as other materials at room temperature was studied by Tiwari et al. (2006). It was found the virus survived on gumboots for three days and no virus was detected on day nine from any of the materials tested. Again the survival time of the virus on gumboots is approximately 30% of the virus’s potential survival time at this temperature. Therefore a uniform distribution assuming a moderate probability of survival on gumboots was also created (Uniform(0.3,0.7)). Nazir et al. (2011) reported some variability in the survivability amongst the different AI virus subtypes. However there is limited scientific literature on such comparisons. Therefore for the purpose of this model, it is assumed the survival of LPAI viruses is similar across the different subtypes.

The distributions from the literature were then multiplied by the corresponding proportion of answers from the survey results. For instance, the Beta distribution created for mice/rats from the literature was multiplied by the proportion of mice/rat answers from the survey results. Similarly, the beta distribution of insects from literature, the uniform distribution of virus survival in duck faeces and the uniform distribution of virus survival on gumboots was multiplied by the proportion of insects, farm cat or dog access and absence of footbath answers from the survey results respectively. All multiplications were then added together to give a single probability per farm type. This probability incorporates both literature and survey results and was used as the input parameter for this node.

**References**

Achenbach, J.E., and Bowen, R.A. (2011). Transmission of Avian Influenza A Viruses among Species in an Artificial Barnyard. *PloS One* 6(3).

Alexander, D., and Capua, I. (2008). Avian influenza in poultry. *World's Poultry Science Journal* 24**,** 513-532.

Alexander, D.J. (2007). An overview of the epidemiology of avian influenza. *Vaccine* 25(30)**,** 5637-5644.

BOM (Bureau of Meterology) (2016). *Weather Station Directory* [Online]. Available: <http://www.bom.gov.au/climate/data/stations/> [Accessed 4 February 2016].

Grillo, V., Arzey, K., Hansbro, P., Hurt, A., Warner, S., Bergfeld, J., et al. (2015). Avian influenza in Australia: a summary of 5 years of wild bird surveillance. *Australian Veterinary Journal* 93(11)**,** 387-393.

Hansbro, P.M., Warner, S., Tracey, J.P., Arzey, K.E., Selleck, P., O'Riley, K., et al. (2010). Surveillance and analysis of avian influenza viruses, Australia. *Emerging Infectious Diseases* 16(12)**,** 1896-1904.

Henzler, D., Kradel, D., Davison, S., Ziegler, A., Singletary, D., DeBok, P., et al. (2003). Epidemiology, Production Losses, and Control Measures Associated with an Outbreak of Avian Influenza Subtype H7N2 in Pennsylvania (1996-98). *Avian Diseases* 47**,** 1022-1036.

Jonges, M., Leuken, J.v., Wouters, I., Koch, G., Meijer, A., and Koopmans, M. (2015). Wind-Mediated Spread of Low Pathogenic Avian Influenza Virus into the Envrionment during OUtbreaks at Commercial Poultry Farms. *PloS One*.

Nazir, J., Haumacher, R., Ike, A.C., and Marschang, R.E. (2011). Persistence of Avian Influenza Viruses in Lake Sediment, Duck Feces, and Duck Meat. *Applied and Environmental Microbiology* 77(14)**,** 4981-4985.

Nielsen, A.A., Sovgard, H., Stockmarr, A., Handberg, K.J., and Jorgensen, P.H. (2011). Persistence of Low-Pathogenic Avian Influenza H5N7 and H7N1 Subtypes in House Flies (Diptera: Muscidae). *Journal of Medical Entomology* 48(3)**,** 608-614.

Olsen, B., Munster, V.J., Wallensten, A., Waldenstrom, J., Osterhause, A.D., and Fouchier, R.A. (2006). Global Patterns of Influenza A Virus in Wild Birds. *Science* 312(5772)**,** 384-388.

Swayne, D.E. (2008). *Avian Influenza.* Iowa USA: Blackwell Publishing.

Tiwari, A., Patnayak, D.P., Chander, Y., Parsad, M., and Goyal, S.M. (2006). Survival of Two Avian Respiratory Viruses on Porous and Nonporous Surfaces. *Avian Diseases* 50(2)**,** 284-287.
